# Overall survival and prognostic factors of oral cavity and oropharyngeal squamous cell carcinomas in Saudi Arabia: a population-based cohort study (2000–2019)

**DOI:** 10.3389/fonc.2026.1821647

**Published:** 2026-06-11

**Authors:** Waad R. Al-Amri, Abdullah S. Al-Swuailem

**Affiliations:** Department of Periodontics and Community Dentistry, College of Dentistry, King Saud University, Riyadh, Saudi Arabia

**Keywords:** oral cavity cancer, oropharyngeal cancer, population-based, Saudi Arabia, squamous cell carcinoma, survival rate

## Abstract

Oral cavity and oropharyngeal squamous cell carcinomas (OC-SCC and OP-SCC) are major contributors to head and neck cancer mortality worldwide. However, long-term population-based survival patterns that separately evaluate these subsites remain limited in Saudi Arabia. This study assessed overall survival (OS) and independent prognostic factors in a nationwide cohort diagnosed between 2000 and 2019. A retrospective population-based cohort study was conducted using data from the Saudi Cancer Registry. The analysis included demographic characteristics (age, sex, region of residence), year of diagnosis, and stage at diagnosis. OS was estimated using the Kaplan–Meier method, and multivariable Cox proportional hazards regression models were used to identify independent predictors of mortality. Statistical analyses were performed using Stata 18. The cohort included 3,114 patients. Five-year OS was 55% for OC-SCC and 49% for OP-SCC. In OC-SCC, survival declined markedly with advancing stage, with 5-year OS decreasing from 65% in localized disease to 38% in distant-stage disease. Patients aged ≥75 years had higher mortality compared with those ≤45 years (aHR 1.70, 95% CI 1.40–2.07), and males had increased risk compared with females (aHR 1.49, 95% CI 1.33–1.66). Diagnosis during 2010–2019 was also associated with higher mortality (aHR 1.37, 95% CI 1.22–1.55). In OP-SCC, regional stage was independently associated with higher mortality compared with localized disease (aHR 1.64, 95% CI 1.08–2.49), and residence in the Western region was associated with increased mortality (aHR 1.62, 95% CI 1.08–2.42). Survival outcomes for OC-SCC and OP-SCC in Saudi Arabia remain modest and vary by subsite and stage at diagnosis. The strong stage-related survival gradient highlights the importance of earlier detection and equitable access to cancer care to improve national outcomes.

## 1 Introduction

Oral cavity and oropharyngeal cancers constitute a major component of the global burden of head and neck cancers and are associated with substantial morbidity and mortality worldwide (1). In 2021, oral cavity cancer (OCC) accounted for an estimated 421,577 new cases and 208,379 deaths globally, underscoring its considerable public health impact (2). Oropharyngeal cancer (OPC), while less common, remains a significant contributor, with approximately 98,400 new cases and 48,100 deaths worldwide in 2020, corresponding to age-standardized incidence and mortality rates of 1.1 and 0.51 per 100,000, respectively (3). These malignancies are predominantly squamous cell carcinoma (SCC), accounting for more than 90% of cases (4, 5). Oral cavity squamous cell carcinoma (OC-SCC) arises from the lips and oral cavity subsites, whereas oropharyngeal squamous cell carcinoma (OP-SCC) originates from the tonsils, base of tongue, and other oropharyngeal structures (4). Despite their histologic similarity, OC-SCC and OP-SCC are now recognized as biologically and clinically distinct entities. OC-SCC is predominantly associated with tobacco and alcohol exposure, with additional regional contributions from areca nut use, while Human Papillomavirus (HPV) plays only a minor etiologic role, accounting for approximately 3% of cases (6). In contrast, OP-SCC in many high-income countries is now predominantly HPV-driven, most commonly by HPV-16 (7). These etiologic differences have important biological and clinical consequences. HPV-positive OP-SCC exhibits unique molecular features, increased radiosensitivity, and substantially better survival compared with HPV-negative disease, whereas OC-SCC remains an aggressive malignancy with limited improvement in long-term survival over recent decades (7–9). Clinically, OC-SCC more often presents as visible mucosal lesions amenable to early detection, whereas OP-SCC frequently presents with small or occult primary tumors and early cervical nodal metastasis (5, 10). The survival outcomes of these two types of cancer are affected by factors related to the population and healthcare context. Historically and in settings with low HPV prevalence, OC-SCC has demonstrated higher OS, reflecting earlier clinical detectability and greater use of primary surgical management, whereas OP-SCC has presented with advanced nodal disease and poorer outcomes (11–13). However, the survival for OP-SCC in higher-income countries has improved markedly with the increasing prevalence of HPV-associated disease, which shows substantially better prognosis than HPV-negative tumors, and in some series, the survival of patients with OP-SCC was comparable to or better than patients with OC-SCC (14–16). Across both sites, stage at diagnosis remains the strongest predictor of prognosis, with early-stage diagnosis associated with favorable survival compared to advanced-stage diagnosis (17–19). However, whether the HPV-associated survival advantage observed in high-income countries applies to Middle Eastern populations with lower HPV prevalence remains unclear. In Saudi Arabia (SA), evidence on OC-SCC and OP-SCC is derived mostly from small, hospital-based cohorts, limiting population-level inference and national generalizability. Data from multiple tertiary-care series in Riyadh, SA consistently showed that OC-SCC presents at an advanced stage, with approximately two-thirds to four-fifths of patients diagnosed at stage III–IV across independent cohorts (20, 21). In these limited institutional reports, the 5-year OS for OC-SCC is approximately 60% with marked stage dependence, where early-stage diagnosis (~77% for stage I–II) has substantially better outcomes compared to advanced stages (~46% for stage III–IV) (20, 22). Older age and cervical lymph node metastasis were also predictors of poorer survival (22). However, these hospital-based estimates are subject to referral bias, stage migration, and incomplete follow-up.

In SA, OP-SCC is relatively uncommon and is rarely analyzed as a distinct subsite. Evidence on HPV-related OP-SCC in SA is limited to a single national referral-center study of head and neck SCC. In this study, the reported overall HPV prevalence was 3.5%, with a higher proportion among OP-SCC (~21%) and a significantly better survival in p16/HPV-positive cases (23). Another Saudi report described an advanced stage at presentation, and a high metastatic burden without providing OPC-specific survival estimates (24). While hospital-based data mirror international findings that stage, nodal involvement, and age are dominant prognostic factors (19, 25–27), national population-based survival estimates for OC-SCC and OP-SCC remain unavailable in SA. To our knowledge, no nationwide population-based study has separately evaluated OS outcomes for OC-SCC and OP-SCC in SA. Therefore, the present study estimated population-based OS for OC-SCC and OP-SCC in SA and assessed the effects of age, sex, region of residence, year of diagnosis, and stage at diagnosis on survival.

## 2 Materials and methods

### 2.1 Study design and data sources

This study is a retrospective population-based cohort study of OC-SCC and OP-SCC patients in SA from 1 January 2000 to 31 December 2019. Cancer cases registration in SA started in 1994, and the data for this study were obtained from the Saudi Cancer Registry (SCR), a national population-based registry operating under the National Cancer Center of the Saudi Health Council (28). The SCR systematically collects information on cancer cases diagnosed in SA through mandatory reporting from healthcare facilities. The data included full dates of birth and diagnosis; last known vital status; administrative region of residence; nationality; tumor morphology, behavior, grade, and stage at diagnosis; and, when available, the date and cause of death, as recorded on death notifications or death certificates. The quality and validity of registry data, including stage information, have been previously evaluated through internal re-abstraction studies, which found them to be highly concordant (29). Vital status and date of death were ascertained through passive follow-up using deterministic linkage of SCR records with mortality data obtained from the National Information Center (NIC) of the Ministry of Interior, based on unique personal identifiers. For cases in which NIC information was unavailable, supplemental follow-up data from SCR were used. Linkage was completed on 24 July 2023, representing the most recent and complete ascertainment of vital status for the study cohort. Because non-Saudi nationals often reside in SA for limited periods and may leave after diagnosis, follow-up of vital status through national linkage is less complete for this population. In addition, deaths occurring abroad may not be captured. Therefore, the OS analyses were restricted to Saudi nationals to minimize bias and improve the validity of survival estimates.

### 2.2 Case definition and study variables

**Figure 1:**
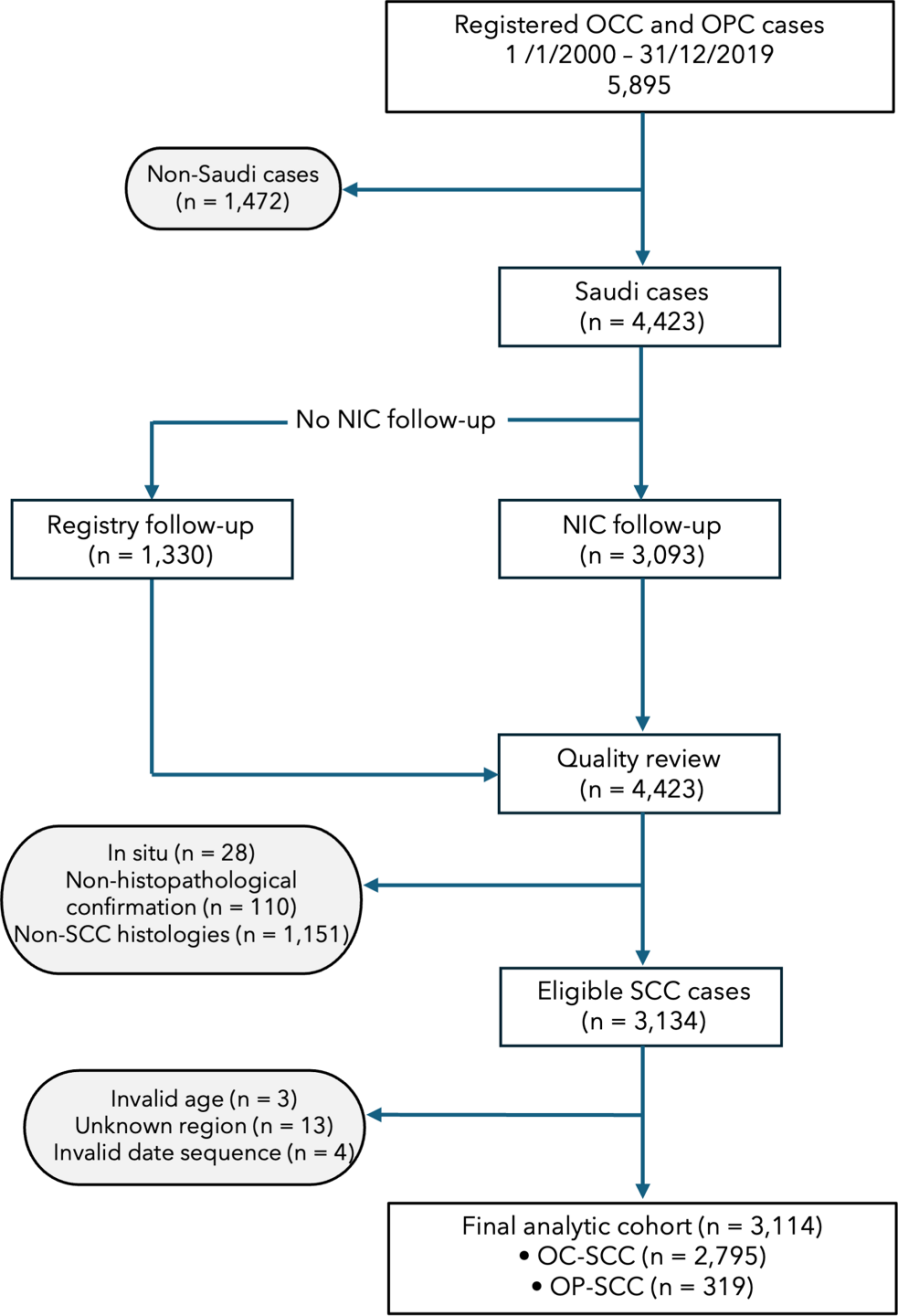
Records included in survival analysis. OCC, oral cavity cancer; OPC, oropharyngeal; NIC, National Information Center; SCC, squamous cell carcinoma.

The study cohort was restricted to Saudi nationals (N = 4,423) diagnosed with primary invasive OCC or OPC, identified using the International Classification of Diseases for Oncology, Third Edition (ICD-O-3) (30). OCC was defined as tumors arising in the internal lip (C00.3–C00.9), oral tongue (C02.0–C02.3, C02.8–C02.9), gingiva (C03), floor of mouth (C04), hard palate and adjacent oral structures (C05.0–C05.9), and other parts of the mouth (C06). OPC included tumors of the base of tongue (C01), tonsil (C09), oropharynx (C10), and other ill-defined oropharyngeal sites (C14.0). Cancers of the external lip (C00.0–C00.2), salivary glands (C07–C08), nasopharynx (C11), and hypopharynx (C12–C13) were excluded due to their distinct clinical and etiological profiles (31, 32). Analysis was limited to cases with confirmed histological diagnosis of SCC based on ICD-O-3 morphology codes (8032, 8033, 8050-8052, 8070-8078, 8082-8084, 8094, and 8123) corresponding to squamous histology and recognized variants. After application of histologic eligibility and data-quality criteria, the final analytic cohort comprised 3,114 SCC cases (**Figure 1**). Age groups were empirically derived using the mean and standard deviation of age at diagnosis across OC-SCC and OP-SCC cases. Across all analytic subsets, mean age was approximately 60 years with a standard deviation of 15–16 years, corresponding to mean ± SD boundaries of approximately 45 and 76 years. These values were rounded to clinically meaningful cut-points, consistent with age thresholds commonly used in prior oral cancer studies (33–42), to define three age groups: (≤45 years, 46–74 years, and ≥75 years), ensuring internal consistency and adequate subgroup sizes across subsites and histological strata. Regional data were harmonized into five macro-regions based on administrative boundaries: Central (Riyadh, Qassim), Eastern, Western (Makkah, Madinah), Northern (Al-Jouf, Northern Borders, Hail, Tabuk), and Southern (Asir, Jazan, Najran, Baha); regions recorded as International or Unknown were grouped under Other/Unknown. Stage at diagnosis was classified according to the Surveillance, Epidemiology, and End Results (SEER Summary Stage 2000) system as: localized (cancer confined to the primary site of origin); regional (including direct extension beyond the primary site and/or involvement of regional lymph nodes); and distant (tumor spread to distant organs or non-regional lymph nodes). Cases in which the extent of disease cannot be determined were classified as unknown stage (43). The diagnosis year was categorized into two calendar periods (2000–2009 and 2010–2019).

### 2.3 Survival data preparation and follow-up

The OS was the primary outcome, defined as the time from histopathological diagnosis to death from any cause. Patients were followed from diagnosis until death or the date of last known vital status, whichever occurred first. Individuals who were alive at the end of follow-up or whose vital status was unknown were treated as censored. Administrative censoring was applied on 24 July 2023, corresponding to the most recent registry–national linkage update available for this study. Patients alive at this date were censored at their last known vital status, ascertained through national linkage with the National Information Center (NIC) or, when unavailable, registry follow-up records. Survival time was calculated in years. To avoid zero survival times, one day was added when the dates of diagnosis and death were identical. No assumptions were made regarding survival beyond the recorded follow-up period. Records with missing diagnosis dates, missing follow-up dates, or implausible temporal sequences (e.g., follow-up preceding diagnosis) were excluded from survival analyses.

### 2.4 Statistical analysis

Baseline characteristics were summarized for the analytic cohort as overall and stratified by tumor subsite (OC-SCC & OP-SCC). Age at diagnosis was reported as the mean (SD). Categorical variables, including age group, sex, region of residence (five macro-regions), diagnosis period (2000–2009 vs 2010–2019), and SEER summary stage (localized, regional, distant, unknown), were summarized as frequencies and column percentages within each subsite. The total number of patients contributing to each category is reported in **Table 1**. The OS probabilities at 1 and 5 years after diagnosis were estimated using the Kaplan–Meier method. Survival curves were generated for the overall cohort and stratified by age group, sex, region of residence, tumor subsite, stage at diagnosis, and period of diagnosis. Differences in survival distributions between groups were assessed using the log-rank test. Median observed survival time, and corresponding 95% confidence intervals (CIs) were reported. Univariate Cox proportional hazards models were first fitted to estimate hazard ratios (HRs) and 95% confidence intervals (CIs) for factors associated with overall mortality. Covariates examined included age group, sex, region of residence, tumor subsite, diagnosis period, and stage at diagnosis. A multivariate Cox proportional hazards model was then constructed, including age group (≤45, 46–74, ≥75 years), sex, region of residence (five macro-regions), and diagnosis period (2000–2009 vs 2010–2019). The proportional hazards assumption was assessed separately for the OC-SCC and OP-SCC multivariable Cox models using Schoenfeld residuals. For OC-SCC, violations were observed for stage at diagnosis and certain regional categories; therefore, a stage-stratified Cox model was applied. Following stratification, the proportional hazards assumption was reassessed for the remaining covariates, confirming that the stratified model adequately addressed the major source of non-proportionality. For OP-SCC, the global test did not indicate a significant violation, and hence a standard Cox model was retained. Detailed results of the proportional hazards assessment before and after stratification are provided in **Supplementary Tables S4A–C**. As a sensitivity analysis for OC-SCC, accelerated failure time (AFT) models (Weibull, log-logistic, and log-normal) were fitted using robust standard errors, with stage included as a covariate. Model fit was compared using Akaike’s Information Criterion (AIC) and Bayesian Information Criterion (BIC). Because AFT models do not support stratification analogous to the Cox model, results were used to assess the robustness and consistency of the primary findings; detailed results are provided in **Supplementary Table 5**. Robust standard errors were used in all Cox models. All statistical analyses were conducted using STATA version 18 (StataCorp, College Station, TX, USA) (44). Statistical significance was assessed at α = 0.05.

**Table 1:** Baseline characteristics of patients with oral cavity and oropharyngeal squamous cell carcinoma diagnosed in Saudi Arabia, 2000–2019.

Characteristic	OC-SCC (n = 2,795)	OP-SCC (n = 319)	Overall (N = 3,114)
Age at diagnosis, years			
Mean ± SD	61.0 ± 15.6	60.5 ± 15.9	61.0 ± 15.6
Age group, n (%)			
≤45 years	456 (16.31)	57 (17.87)	513 (16.47)
46–74 years	1,787 (63.94)	199 (62.38)	1,986 (63.78)
≥75 years	552 (19.75)	63 (19.75)	615 (19.75)
Sex, n (%)			
Female	1,351 (48.34)	146 (45.77)	1,497 (48.07)
Male	1,444 (51.66)	173 (54.23)	1,617 (51.93)
Region of residence, n (%)			
Central SA	562 (20.11)	86 (26.96)	648 (20.81)
Western SA	710 (25.40)	110 (34.48)	820 (26.33)
Eastern SA	315 (11.27)	40 (12.54)	355 (11.40)
Southern SA	1,071 (38.32)	58 (18.18)	1,129 (36.26)
Northern SA	137 (4.90)	25 (7.84)	162 (5.20)
Diagnosis period, n (%)			
2000–2009	1,113 (39.82)	142 (44.51)	1,255 (40.30)
2010–2019	1,682 (60.18)	177 (55.49)	1,859 (59.70)
Stage at diagnosis (SEER), n (%)			
Localized	1,123 (40.18)	88 (27.59)	1,211 (38.89)
Regional	1,110 (39.71)	148 (46.39)	1,258 (40.40)
Distant	314 (11.23)	50 (15.67)	364 (11.69)
Unknown	248 (8.87)	33 (10.34)	281 (9.02)
Vital status at end of follow-up, n (%)			
Alive	1,213 (43.40)	118 (36.99)	1,331 (42.74)
Dead	1,266 (45.30)	156 (48.90)	1,422 (45.66)
Unknown	316 (11.31)	45 (14.11)	361 (11.59)
5-year follow-up outcome*, n (%)			
Death ≤5 years	1,083 (38.75)	140 (43.89)	1,223 (39.27)
Censored at 5 years	1,712 (61.25)	179 (56.11)	1,891 (60.73)

OC-SCC, oral cavity squamous cell carcinoma; OP-SCC, oropharyngeal squamous cell carcinoma; SD, standard deviation. * The 5-year follow-up outcome was defined based on observed OS within 5 years from diagnosis. “Death ≤5 years” indicates patients who died within 5 years of diagnosis. “Censored at 5 years” includes patients who were alive at 5 years or censored before 5 years due to administrative censoring (24 July 2023) or loss to follow-up.

## 3 Results

### 3.1 Study population and baseline characteristics

The cohort included 3,114 patients with OC-SCC and OP-SCC diagnosed between 2000 and 2019 in SA. The vast majority of cases were OC-SCC (n = 2,795; 89.8%), whereas 319 cases (10.2%) were OP-SCC. The mean age at diagnosis was 61.0 ± 15.6 years, with similar age distributions among OC-SCC and OP-SCC cases. Nearly two-thirds of patients (63.8%) were diagnosed between ages 46 and 74, while 16.5% were 45 years or younger and 19.8% were 75 years or older at diagnosis. Approximately half of patients were male (51.9%), with a higher proportion of males in OP-SCC than in OC-SCC (54.2% vs 51.7%). Geographic distribution varied by subsite. While more than one-third of the OC-SCC cases were diagnosed in the Southern region (38.3%), OP-SCC cases were more commonly diagnosed in the Western region (34.5%) and Central region (27.0%). Most diagnoses for OC-SCC and OP-SCC occurred during 2010–2019 (60.2%, 55.5%) compared to 2000-2009 (39.8%, 44.5%), respectively. At diagnosis, 38.9% of patients had localized disease, 40.4% regional disease, 11.7% distant disease, and 9.0% had unknown stage. OP-SCC patients had a higher proportion of regional (46.4%) and distant-stage (15.7%) disease than OC-SCC patients. During the entire follow-up period, 45.7% of patients were recorded as deceased (**Table 1**). Within 5 years, 39.3% of the cohort died; mortality was higher in OP-SCC than OC-SCC (43.9% vs. 38.8%).

### 3.2 Overall survival by cancer subsite

**Figure 2:**
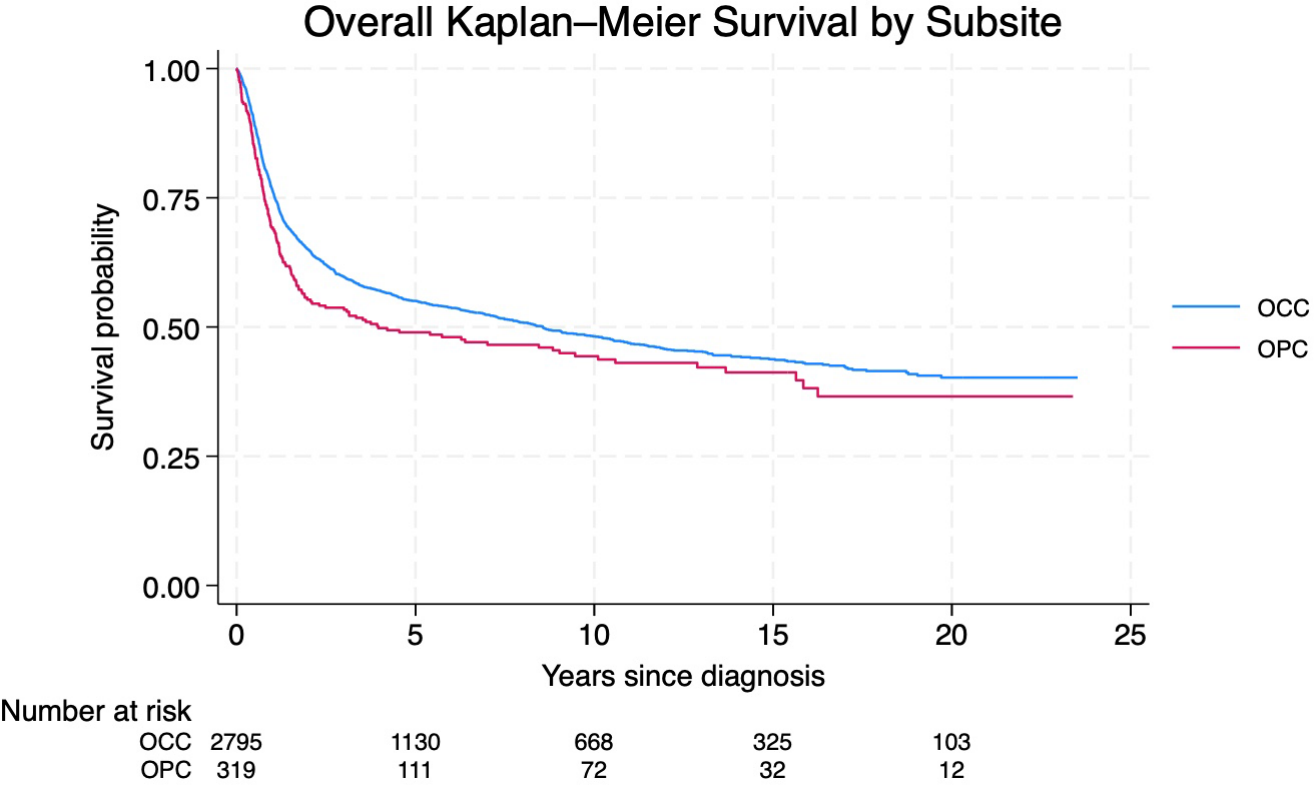
Overall Kaplan–Meier survival by cancer subsite among Saudi patients diagnosed with oral cavity squamous cell carcinoma (OC-SCC) and oropharyngeal squamous cell carcinoma (OP-SCC), 2000–2019.

The median follow-up time was 9.88 years (IQR: 4.67–15.25), corresponding to 17,365.9 person-years of observation. Median follow-up was similar across subsites (9.86 years for OC-SCC and 10.04 years for OP-SCC). Kaplan–Meier curves showed higher OS in OC-SCC than in OP-SCC, with an early separation that narrowed over time (**Figure 2**). The median survival time for OC-SCC was 8.55 years, with 1-year OS of 76% and 5-year OS of 55%. In contrast, OP-SCC patients had a median survival of 3.95 years, with corresponding 1-year and 5-year OS rates of 69% and 49%, respectively (**Supplementary Tables S1, S2**).

### 3.3 Survival outcomes in oral cavity squamous cell carcinoma

#### 3.3.1 Kaplan–Meier survival estimates

**Figure 3:**
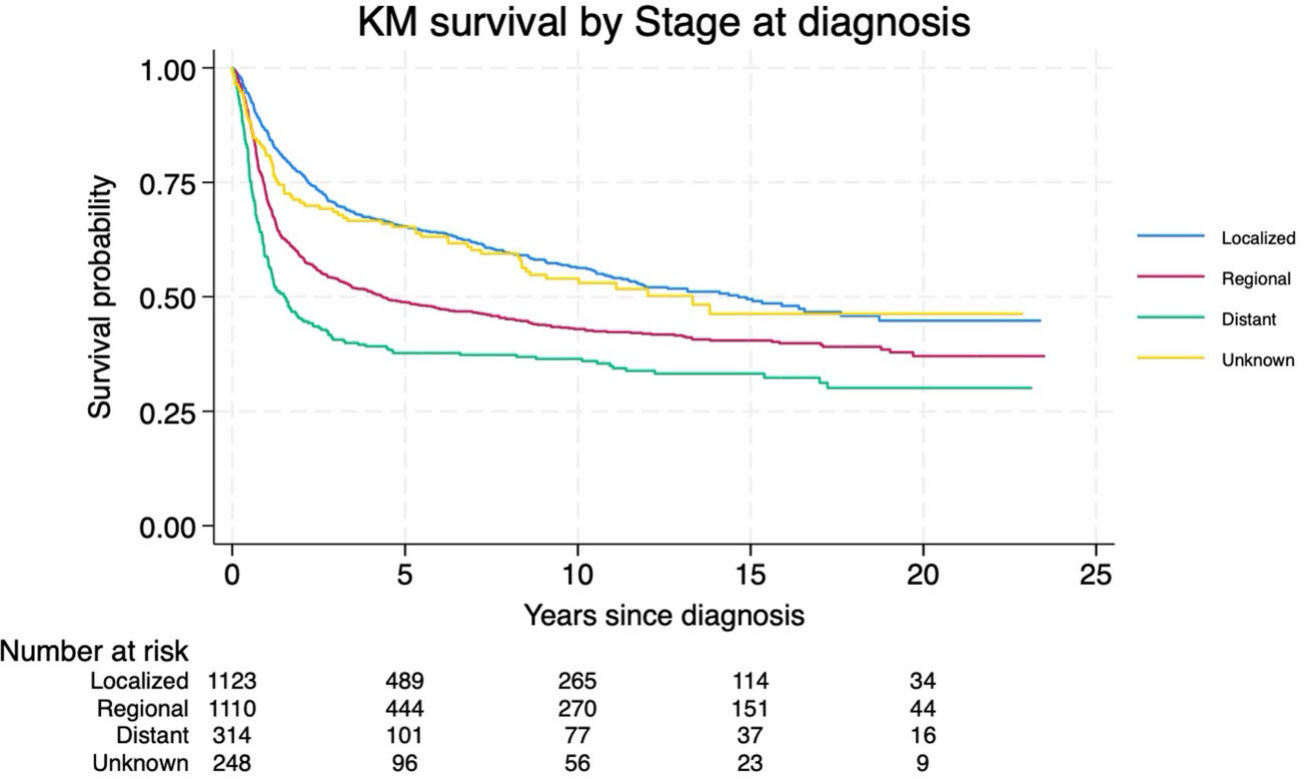
Kaplan–Meier survival curves by SEER stage at diagnosis among Saudi patients with oral cavity squamous cell carcinoma (2000–2019).

Among OC-SCC patients, survival varied significantly by age, sex, region, stage at diagnosis, and diagnosis period (**Supplementary Table 1**; **Supplementary Figures 1–4**). Younger (≤45 years) and middle-aged (46–74 years) patients had similar survival, with 5-year OS of 61% in both groups, whereas patients aged ≥75 years had substantially poorer outcomes, with a 5-year OS of 49% (p < 0.001). Female OC-SCC patients had a higher 5-year OS rate than male patients (61% vs. 49%, p < 0.001). Survival differed modestly by region (p = 0.041). Five-year OS was broadly comparable across the Central, Eastern, and Southern regions (~60%), whereas lower survival was observed in the Northern and Western regions (~50%). Stage at diagnosis was strongly associated with survival (p < 0.001). Patients diagnosed with localized stage had the most favorable outcomes, with a 5-year OS of 65%, compared with 49% for regional stage and 38% for distant stage (**Figure 3**). Furthermore, patients diagnosed during 2010–2019 had lower 5-year OS than those diagnosed during 2000–2009 (51% vs. 62%; p < 0.001).

#### 3.3.2 Multivariate Cox regression analysis

**Figure 4:**
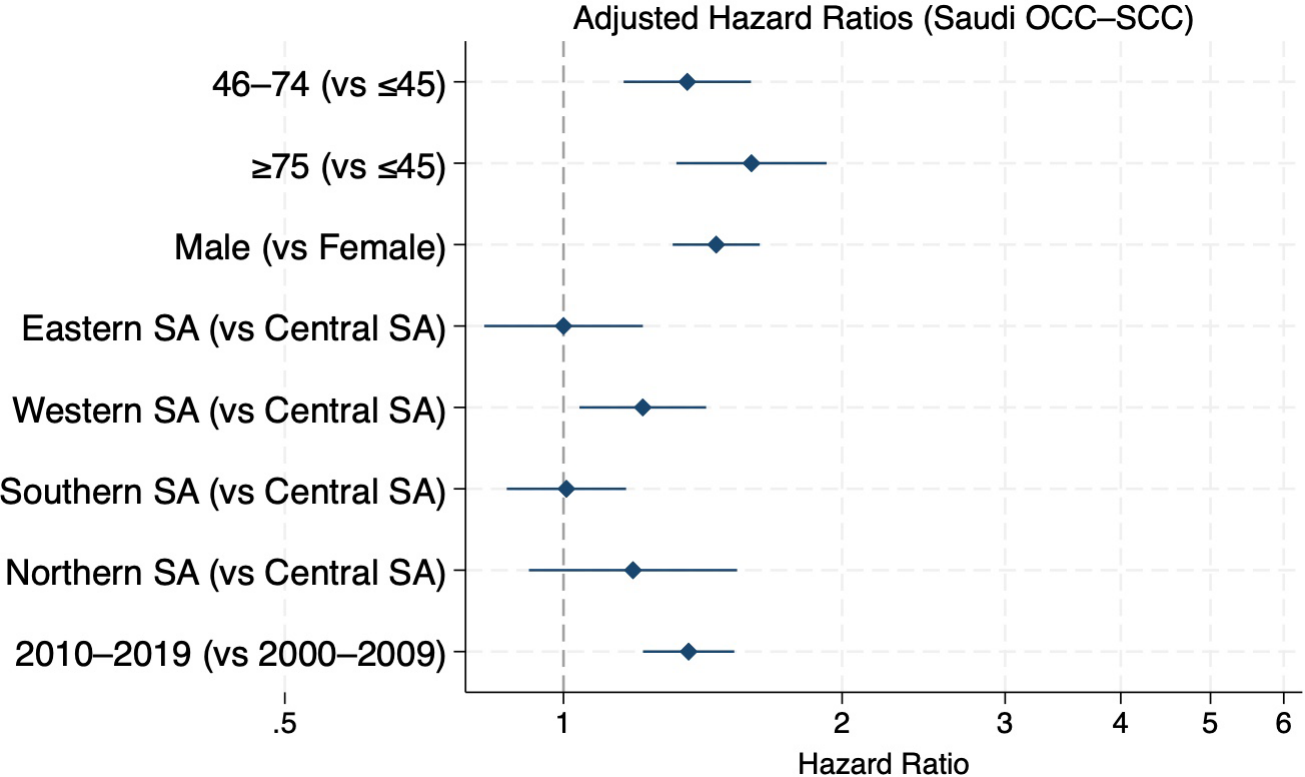
Adjusted hazard ratios for overall mortality among Saudi patients with oral cavity squamous cell carcinoma. Forest plot showing adjusted hazard ratios (aHRs) and 95% confidence intervals from a Cox proportional hazards model stratified by SEER stage at diagnosis. The model was adjusted for age group, sex, region, and diagnosis period. The dashed vertical line indicates the null value (HR = 1.0). Hazard ratios greater than 1 indicate an increased risk of mortality relative to the reference category.

In stage-stratified Cox proportional hazards models (**Table 2**; **Figure 4**), mortality risk significantly increased with age. Compared with patients aged 45 years and younger, patients aged 46–74 years and patients aged 75 years and older had significantly higher adjusted hazard ratios (aHR: 1.44; 95% CI: 1.22–1.70; p < 0.001) and (aHR: 1.70; 95% CI: 1.40–2.07; p < 0.001), respectively. Male patients were associated with a statistically higher risk of death compared to female patients (aHR: 1.49; 95% CI: 1.33–1.66; p < 0.001). Diagnosis during 2010–2019 was independently associated with higher mortality than during 2000–2009 (aHR: 1.37; 95% CI: 1.22–1.55; p < 0.001). After multivariate adjustment, region of residence was not significantly associated with mortality, although the Northern region showed a non-significant trend toward increased risk (aHR: 1.26; 95% CI: 0.96–1.65; p = 0.099). Results from the unstratified Cox proportional hazards regression model were consistent with the stratified analysis (**Supplementary Table 3**). In the unstratified model, regional and distant stages were associated with significantly higher hazards of death compared with localized stage (aHR = 1.6, 95% CI = 1.42–1.83; and aHR = 2.2, 95% CI = 1.84–2.67, both p < 0.001), while cases with unknown stage had no significant difference in mortality (aHR = 1.14, 95% CI = 0.88–1.46: p = 0.321). Assessment of proportional hazards assumptions using Schoenfeld residuals confirmed violation of the proportional hazards assumption in the unstratified OC-SCC model, justifying the use of stage-stratified Cox regression; detailed results are provided in **Supplementary Tables 4A, B**. Sensitivity analyses using accelerated failure time (AFT) models for OC-SCC showed consistent directional findings with the stage-stratified Cox model, with the log-normal model providing the best fit (**Supplementary Table 5**).

**Table 2:** Stage-stratified Cox proportional hazards regression for overall mortality among Saudi patients with oral cavity squamous cell carcinoma (2000–2019).

Variable	Unadjusted HR (95% CI)	p-value	Adjusted HR (95% CI)	p-value
Age group				
≤45 years (ref)	1.00	—	1.00	—
46–74 years	1.38 (1.17–1.63)	<0.001	1.44 (1.22–1.70)	<0.001
≥75 years	1.62 (1.33–1.97)	<0.001	1.70 (1.40–2.07)	<0.001
Sex				
Female (ref)	1.00	—	1.00	—
Male	1.47 (1.32–1.65)	<0.001	1.49 (1.33–1.66)	<0.001
Region of residence				
Central SA (ref)	1.00	—	1.00	—
Eastern SA	0.96 (0.77–1.19)	0.713	0.96 (0.78–1.19)	0.705
Western SA	1.16 (0.98–1.37)	0.079	1.14 (0.96–1.35)	0.132
Southern SA	1.03 (0.88–1.20)	0.743	1.00 (0.85–1.17)	0.996
Northern SA	1.37 (1.05–1.78)	0.021	1.26 (0.96–1.65)	0.099
Diagnosis period				
2000–2009 (ref)	1.00	—	1.00	—
2010–2019	1.31 (1.16–1.47)	<0.001	1.37 (1.22–1.55)	<0.001

Hazard ratios (HRs) and 95% confidence intervals (CIs) were estimated using Cox proportional hazards regression stratified by SEER summary stage at diagnosis due to violation of the proportional hazards assumption. Multivariate models were adjusted for age group, sex, region of residence, and diagnosis period. HR > 1 indicates higher hazard of death relative to the reference category.

### 3.4 Survival outcomes in oropharyngeal squamous cell carcinoma

#### 3.4.1 Kaplan–Meier survival estimates

**Figure 5:**
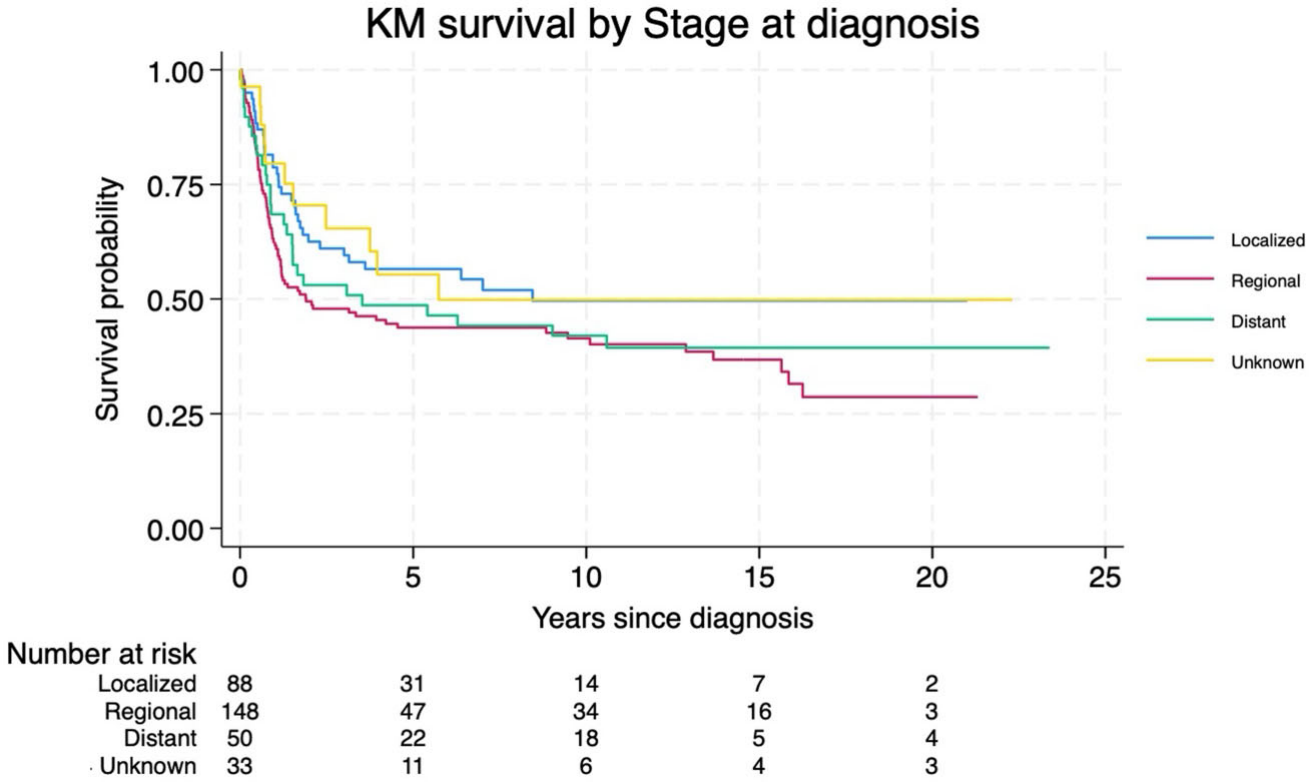
Kaplan–Meier survival curves stratified by SEER summary stage at diagnosis among Saudi patients with oropharyngeal squamous cell carcinoma, 2000–2019.

In contrast to OC-SCC, survival differences in OP-SCC were less pronounced across different demographic groups (**Supplementary Table 2**; **Supplementary Figures 4–8**). The 5-year OS rate for OP-SCC was 49%, with no statistically significant differences in survival between male and female patients or across age groups (p = 0.148). Survival also did not differ significantly by region of residence (p = 0.102). However, 5-year OS was lower in the Western region (39%) than in the Central (55%) and Southern (58%) regions. Five-year OS ranged from 44% to 57% across SEER stages (p = 0.148) and declined from 55% in 2000–2009 to 45% in 2010–2019 (p = 0.117) (**Figure 5**).

#### 3.4.2 Multivariate Cox regression model analysis

**Figure 6:**
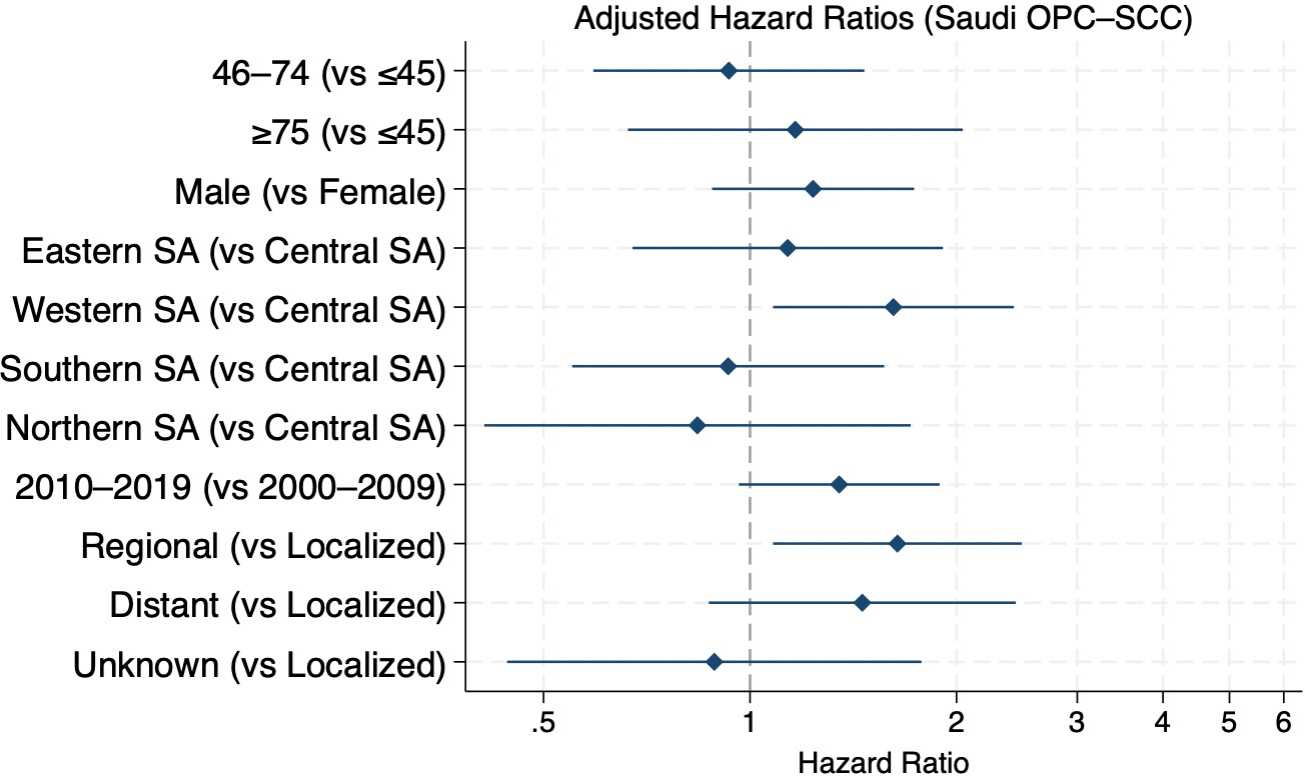
Forest plot of adjusted hazard ratios (HRs) for overall mortality among Saudi patients with oropharyngeal squamous cell carcinoma. Estimates derived from multivariate Cox proportional hazards regression adjusted for age group, sex, region, diagnosis period, and stage at diagnosis. Error bars indicate 95% confidence intervals.

In multivariate Cox regression (**Table 3**; **Figure 6**), stage at diagnosis emerged as the strongest predictor of mortality. Compared with localized stage, regional stage was associated with a significantly higher risk of death (aHR: 1.64; 95% CI: 1.08–2.49; p = 0.020) while distant stage showed a non-significant trend toward higher mortality. Residence in Western region was independently associated with increased mortality compared to those in Central region (aHR: 1.62; 95% CI: 1.08–2.42; p = 0.020). Later diagnosis period (2010–2019) was associated with a non-significant trend toward higher risk of death (aHR: 1.35; 95% CI: 0.96–1.89; p = 0.082). Age group and sex variables were not statistically significant predictors after adjustment, with aHRs close to unity and wide confidence intervals.

**Table 3:** Cox proportional hazards regression for overall mortality among Saudi patients with oropharyngeal squamous cell carcinoma (2000–2019).

Variable	Unadjusted HR (95% CI)	p-value	Adjusted HR (95% CI)	p-value
Age group				
≤45 years (Reference)	1.00	—	1.00	—
46–74 years	0.91 (0.59–1.41)	0.684	0.93 (0.59–1.47)	0.760
≥75 years	1.14 (0.67–1.97)	0.625	1.16 (0.66–2.04)	0.596
Sex				
Female (Reference)	1.00	—	1.00	—
Male	1.27 (0.92–1.74)	0.151	1.24 (0.88–1.73)	0.222
Region of residence				
Central SA (Reference)	1.00	—	1.00	—
Eastern SA	1.22 (0.73–2.03)	0.454	1.13 (0.67–1.91)	0.635
Western SA	1.48 (1.00–2.18)	0.047	1.62 (1.08–2.42)	0.020
Southern SA	0.84 (0.51–1.39)	0.507	0.93 (0.55–1.57)	0.783
Northern SA	0.88 (0.44–1.77)	0.723	0.84 (0.41–1.71)	0.629
Diagnosis period				
2000–2009 (Reference)	1.00	—	1.00	—
2010–2019	1.30 (0.94–1.81)	0.116	1.35 (0.96–1.89)	0.082
Stage at diagnosis (SEER)				
Localized (Reference)	1.00	—	1.00	—
Regional	1.51 (1.02–2.25)	0.040	1.64 (1.08–2.49)	0.020
Distant	1.34 (0.81–2.20)	0.254	1.46 (0.87–2.44)	0.152
Unknown	0.96 (0.50–1.84)	0.892	0.89 (0.44–1.78)	0.735

Hazard ratios (HRs) and 95% confidence intervals (CIs) were estimated using Cox proportional hazards regression. The multivariate model included age group, sex, region of residence, diagnosis period, and SEER stage at diagnosis. HR > 1 indicates higher hazard of death relative to the reference category.

## 4 Discussion

This large, population-based study provides the first survival analysis of OC-SCC and OP-SCC in SA over two decades. Using linked cancer registry and mortality data from 3,114 Saudi nationals diagnosed between 2000 and 2019, we identified distinct survival patterns and prognostic profiles for OC-SCC and OP-SCC. These findings contribute to the epidemiologic characterization of site-specific oral cancers in SA and provide population-level evidence that may inform cancer control planning and prioritization of early detection strategies.

### 4.1 Overall survival and subsite disparities

Our cohort demonstrated a 5-year OS of 55% for OC-SCC and 49% for OP-SCC, reflecting a substantial disease burden and persistent prognostic challenges in both subsites within the Saudi population. These outcomes fall within a wide range reported globally for head and neck cancers (approximately 28–67% for 5-year OS, depending on region and subsite) (45–47). Prior to this analysis, survival data for OC-SCC and OP-SCC in SA were largely limited to single-institution series, primarily from Riyadh, SA. Our population-based results both support and clarify earlier reported findings. For example, Ashraf et al. (2018) reported that the 5-year OS for OCC in a tertiary center in Riyadh was approximately 60%, with marked stage dependence (~77% for stage I–II versus ~46% for stage III–IV), and these findings largely represent OC-SCC outcomes since ~90% of their cohort were SCC (20). Similarly, AlMadan et al. (2025) reported that the 5-year OS of, 88 patients with oral SCC treated at a military hospital in Riyadh was approximately 60% (22). However, these estimates remain slightly below survival observed in several high-income settings, where 5-year OS for OC-SCC typically ranges between 56–65% (19, 25, 48) and HPV-positive OP-SCC often exceeds 75% (49, 50). While survival outcomes for OC-SCC in Saudi Arabia are broadly comparable to several high-income registry-based cohorts, the lower survival observed in SA for OP-SCC compared to HPV-stratified Western populations could be explained by differences in stage distribution, HPV prevalence, and treatment patterns. The lower survival among OP-SCC patients in SA highlights opportunities for identifying risk factors and improving early cancer detection.

### 4.2 OC-SCC vs. OP-SCC survival and the impact of HPV

As discussed previously, OC-SCC and OP-SCC represent biologically distinct disease entities. In HPV-predominant populations, OP-SCC is associated with substantially improved survival compared with OC-SCC, largely attributable to the favorable prognosis of HPV-positive disease. However, HPV status was not available in this registry-based analysis, precluding direct assessment of its effect on survival in the present cohort (7). Therefore, interpretations related to HPV should be considered contextual and not derived directly from the present dataset. Our data showed that OP-SCC survival was relatively low, with a 5-year OS of 49%, and differed only modestly from OC-SCC (55%). This limited survival difference may be consistent with the absence of a marked HPV-related survival advantage, rather than temporal improvements in OP-SCC outcomes. The findings of this study are consistent with the epidemiologic context of oral cancer in SA, where HPV prevalence in OP-SCC remains relatively low. Available institutional and regional studies, based on heterogeneous testing practices, suggest HPV DNA is detectable in only approximately 3.5% of all head and neck SCC cases and in about 21% of tested OPC in SA, which is substantially lower than the 40–50% HPV prevalence reported in Western populations (23). In high-income countries, the shift toward HPV-driven OP-SCC has led to significant improvements in survival, with OP-SCC outcomes now comparable to or better than those of OC-SCC. For example, recent UK and European data reported a 5-year OS of 60–70% for OP-SCC, compared with 50–60% for OC-SCC (48, 51–53). Similarly, a landmark Phase III Radiation Therapy Oncology Group trial conducted at MD Anderson Cancer Center (Texas, USA) demonstrated significantly superior outcomes among patients with HPV-positive stage III–IV OPC. In this trial, 3-year OS was 82.4% versus 57.1%, and progression-free survival was 73.7% versus 43.4%, compared with HPV-negative OP-SCC (54). In contrast, the observed poor OP-SCC outcomes in our cohort may be consistent with a higher proportion of HPV-unrelated, tobacco-associated disease, as suggested by institutional reports from the region (22).

### 4.3 Age, sex, and stage as dominant prognostic factors

In the multivariate analysis, older age and being a male patient were independently associated with higher mortality in OC-SCC, consistent with established prognostic patterns. Patients aged 75 years and older experienced approximately 70% higher adjusted mortality compared with those aged 45 years or younger. These findings likely reflect age-related frailty, comorbidity burden, and treatment limitations in elderly cancer populations. Supporting this interpretation, a Brazilian study identified age older than 60 years as one of the strongest predictors of poor OS in oral cancer (18). Male patients had a 49% increase in mortality compared to female patients, a finding consistently reported across oral cancer cohorts (19). This higher risk for OC-SCC among male patients in SA could be explained by higher exposure to established carcinogens and sex-based differences in healthcare-seeking behavior, treatment adherence, and underlying biological susceptibility (18). In contrast, sex was not a statistically significant predictor of mortality in OP-SCC after multivariate adjustment (aHR 1.24, 95% CI 0.88–1.73), possibly due to limited statistical power given the small OP-SCC cohort size (n = 319, 156 deaths). Stage at diagnosis was the strongest determinant of survival across both subsites, consistent with published literature worldwide. Patients diagnosed at a localized stage experienced substantially higher 5-year OS (approximately 65% for OC-SCC) than those presenting with regional (~49%) or distant stage (~38%). This significant decline in survival mirrors global data, where it has been reported that early-stage head and neck cancers have a 5-year OS rate of 70–80%, compared to 20% in advanced-stage diagnosis (14). In our study, more than half of the cases (around 52%) presented with regional or metastatic disease. Although this proportion is comparable to, or slightly better than, some hospital-based reports in SA, the high proportion of advanced stages remains suboptimal. Likewise, a major Brazilian cancer center reported III–IV stage in 59% of OC-SCC and 90% of OP-SCC cases at diagnosis (17). Similar survival levels have been reported in hospital-based cohorts from India, where 5-year OS approximates 50%, with nodal involvement and advanced tumor stage strongly influencing prognosis (27). Our registry-based stage distribution (approximately 39% localized, 40% regional, 12% distant, and 9% unknown) indicates a lower proportion of advanced-stage cancer than in large hospital-based cohorts, in which stage III–IV tumors account for approximately 75–80% of cases (55). This distribution likely reflects case-mix concentration in tertiary referral centers. In a population-based registry, the SCR captures cases across all levels of care, including patients who may not be referred to tertiary centers, thereby reducing referral-related selection bias and providing a more representative estimate of stage at diagnosis at the national level. These data offer important baseline evidence for evaluating stage distribution patterns in oral cancer at the population level in SA. Geographic heterogeneity in survival was observed in both Kaplan–Meier stratification and multivariate models. For OC-SCC, the 5-year OS was lower in the Northern and Western regions (49% and 51%) and higher in the Central and Southern regions (56% and 57%). For OP-SCC, residence in the Western region was associated with a 62% higher risk of mortality compared with the Central region (aHR = 1.62). These regional disparities may reflect differences in referral pathways and concentration of tertiary-care oncology services in major urban centers such as Riyadh and Jeddah.

### 4.4 Temporal trends and healthcare system evolution

It is worth mentioning that approximately 60% of cases in our cohort were diagnosed during 2010–2019, compared with 40% during 2000–2009. Similar increases in registered case counts have been reported in SCR–based analyses of several major cancers, including breast, colorectal, lymphoma, lung, and pancreatic cancers, and are consistently attributed to population growth and aging, expanded access to diagnostic and screening services, and improvements in registry completeness, rather than site-specific changes in underlying disease risk (56–61). A notable and concerning finding in our analysis was the lack of improvement in survival and the potential deterioration over the reported two decades. Patients diagnosed in 2010–2019 experienced significantly lower OS in OC-SCC compared with those diagnosed in 2000–2009 (adjusted HR 1.37, p < 0.001), with a similar but non-significant trend observed for OP-SCC. This pattern should be interpreted cautiously, as earlier periods may have been affected by underdiagnosis and limited access to diagnostic services, whereas improved case ascertainment in later years may have increased the capture of patients with advanced stage, contributing to an apparent decline in survival. In contrast, survival gains for OC-SCC and OP-SCC in high-income settings have generally been incremental rather than dramatic. Population-based SEER analyses demonstrate significant improvement over recent decades for oral tongue SCC and OP-SCC, while other head and neck subsites showed minimal changes (62). Similarly, a large Brazilian tertiary cancer center reported only a modest increase in 5-year OS for OP-SCC (45.0%-49.1%) between the early and late 2000s, with OC-SCC survival remaining stable at approximately 52% (17). National registry data from Ireland showed a 5-year disease-specific survival of 58.6% OC-SCC, with gradual improvement over two decades, particularly for tongue tumors (63). Likewise, a 39-year Spanish tertiary-center series reported a limited increase in OC-SCC 5-year OS (62.7% to 71.7%), while recurrence rates remained high (53). In summary, the absence of meaningful population-level improvement in OC-SCC and OP-SCC survival in SA over the past two decades highlights the need to strengthen early detection, ensure equitable access to modern treatment modalities, and reinforce national oral cancer prevention and control strategies.

### 4.5 Methodologic considerations and limitations

This study has several limitations that should be acknowledged. First, we lacked information on treatment modality, treatment quality, recurrence, and comorbidities, which are important mediators of survival. Cause-of-death data were incomplete; therefore, OS rather than cancer-specific survival was estimated, which may be influenced by competing mortality, particularly in older patients. Second, survival estimation was constrained by limitations inherent to registry-based follow-up and death ascertainment. Although linkage to national mortality records was available, incomplete updating of dates of last contact and delayed death registration may have occurred, particularly for earlier diagnostic cohorts with longer follow-up time. Such limitations can inflate survival estimates in earlier periods and exaggerate apparent survival deterioration in more recent cohorts, a phenomenon well documented in Saudi Arabia and the broader Eastern Mediterranean and Gulf Cooperation Council region (64, 65). Accordingly, temporal differences in OC-SCC and OP-SCC survival should be interpreted cautiously, as incomplete follow-up and delayed death registration may lead to overestimation of survival probabilities. Third, restricting the cohort to Saudi nationals improved follow-up completeness and minimized bias related to relocation to other countries; however, this approach limits generalizability to the entire population in SA. However, focusing on nationals provides a more appropriate assessment of the national health system’s performance and avoids differential loss to follow-up. Fourth, HPV status was not recorded in SCR, preventing stratification of OP-SCC by HPV status, an important prognostic factor. Although regional studies report relatively low HPV prevalence in OP-SCC, HPV status was not available in this dataset; therefore, its contribution to survival differences cannot be directly assessed. Future linkage with pathology data (e.g., p16 immunohistochemistry or HPV DNA/RNA testing) would substantially enhance etiologic and prognostic interpretation. Fifth, about 9% of cases had an unknown stage in the registry. Rather than excluding these patients, we retained them as a separate category to avoid selection bias. Interestingly, OC-SCC patients with unknown stage had survival outcomes similar to those with localized stage in our data, suggesting that many of these cases may have had early-stage tumors with incomplete documentation. Nevertheless, residual stage misclassification remains possible. Additionally, SEER summary stage lacks the granularity of tumor, node, metastasis (TNM) staging and may obscure prognostic heterogeneity within stage categories Although stage-stratified Cox models addressed the primary violation of the proportional hazards assumption, minor residual non-proportionality was observed for the Southern region variable; however, this factor was not a primary exposure of interest and its effect estimate was close to null. Finally, the relatively small number of OP-SCC cases limited statistical power for OPC-specific subgroup analyses and may explain the absence of statistically significant associations for some covariates despite point estimates consistent with those observed in OC-SCC. Despite these limitations, the study’s strengths include a large population-based cohort spanning 20 years, a near-complete capture of diagnosed cancers through a mandatory reporting system, and robust linkage to mortality records. These data provide a credible national baseline for monitoring survival trends in OC-SCC and OP-SCC and for evaluating the impact of future public health and clinical interventions. Further strengthening of routinely collected clinical variables within national cancer surveillance systems would allow more refined etiologic interpretation and prognostic modeling of oral cancers in Saudi Arabia.

## 5 Conclusions

This nationwide population-based analysis establishes survival benchmarks for OC-SCC and OP-SCC in Saudi Arabia and confirms that stage at diagnosis is the dominant determinant of prognosis. The absence of clear improvements in survival over two decades and the presence of regional disparities highlight critical opportunities to strengthen early detection and cancer care delivery. These findings provide a foundation for evaluating future interventions to improve oral cancer outcomes in Saudi Arabia.

## Data Availability

The dataset was obtained from the Saudi Cancer Registry under institutional approval. Access to the data is subject to legal, ethical, and regulatory restrictions imposed by the Saudi Health Council. The data are not publicly available and may be accessed only with formal approval from the Saudi Health Council. Requests to access these datasets should be directed to Saudi Health Council – National Health Registry & Surveillance Platform (NHRSP)(https://nhrsp.shc.gov.sa/home/default).
