# Supplementary material for: Overall survival and prognostic factors of oral cavity and oropharyngeal squamous cell carcinomas in Saudi Arabia: a population-based cohort study (2000–2019)
Source: Front Oncol. 2026 Jun 11;16:1821647. doi: 10.3389/fonc.2026.1821647 (PMC13293804; doi:10.3389/fonc.2026.1821647)
Supplement: Supplementary file 1 [file DataSheet1.docx]

Supplementary Material

# Supplementary Figures and Tables

## Supplementary Tables

**Supplementary Table 1.** Kaplan–Meier 1- and 5-year survival estimates for Saudi patients with oral cavity squamous cell carcinoma (2000–2019)

| **Variable** | **Median survival (years, 95% CI)** | **1-year survival (95% CI)** | **5-year survival (95% CI)** | **N** | **Log-rank p** |
| --- | --- | --- | --- | --- | --- |
| Overall | 8.55 (7.24–10.22) | 0.76 (0.75–0.78) | 0.55 (0.53–0.57) | 2,795 | — |
| **Age group** |  |  |  |  | <0.001 |
| ≤45 years | NR (14.89–.) | 0.85 (0.81–0.88) | 0.61 (0.56–0.66) | 456 |  |
| 46–74 years | 8.34 (6.94–10.45) | 0.76 (0.74–0.78) | 0.55 (0.53–0.58) | 1,787 |  |
| ≥75 years | 4.52 (2.76–7.17) | 0.70 (0.66–0.74) | 0.49 (0.44–0.53) | 552 |  |
| **Sex** |  |  |  |  | <0.001 |
| Female | 16.99 (11.73–.) | 0.79 (0.77–0.81) | 0.61 (0.58–0.64) | 1,351 |  |
| Male | 4.52 (3.27–6.12) | 0.74 (0.71–0.76) | 0.49 (0.47–0.52) | 1,444 |  |
| **Region** |  |  |  |  | 0.041 |
| Central SA | 10.55 (7.05–.) | 0.74 (0.70–0.78) | 0.56 (0.52–0.61) | 562 |  |
| Eastern SA | 11.95 (6.39–.) | 0.78 (0.73–0.82) | 0.58 (0.52–0.64) | 315 |  |
| Northern SA | 4.03 (1.90–8.57) | 0.71 (0.62–0.78) | 0.49 (0.39–0.58) | 137 |  |
| Southern SA | 9.03 (7.29–11.02) | 0.79 (0.76–0.81) | 0.57 (0.54–0.60) | 1,071 |  |
| Western SA | 5.90 (3.43–10.36) | 0.75 (0.71–0.78) | 0.51 (0.47–0.55) | 710 |  |
| **Stage at diagnosis (SEER)** |  |  |  |  | <0.001 |
| Localized | 14.64 (11.45–18.72) | 0.86 (0.84–0.88) | 0.65 (0.62–0.68) | 1,123 |  |
| Regional | 4.35 (3.27–6.29) | 0.72 (0.69–0.74) | 0.49 (0.46–0.52) | 1,110 |  |
| Distant | 1.50 (1.13–2.20) | 0.59 (0.53–0.64) | 0.38 (0.32–0.43) | 314 |  |
| Unknown | 13.33 (8.34–.) | 0.81 (0.74–0.86) | 0.65 (0.57–0.72) | 248 |  |
| **Diagnosis period** |  |  |  |  | <0.001 |
| 2000–2009 | 13.83 (10.82–18.72) | 0.79 (0.76–0.81) | 0.62 (0.58–0.65) | 1,113 |  |
| 2010–2019 | 6.17 (4.44–7.74) | 0.75 (0.73–0.77) | 0.51 (0.49–0.54) | 1,682 |  |

**Notes.** Median survival times represent median observed survival in years with 95% confidence intervals. Survival probabilities were estimated using the Kaplan–Meier method. Log-rank tests were used to compare groups.. OCC, oral cavity squamous cell carcinoma; OPC, oropharyngeal squamous cell carcinoma; CI, confidence interval. NR = median not reached (upper CI not estimable in Stata output, shown as “.”).

**Supplementary Table 2.** Kaplan–Meier survival estimates for Saudi patients with oropharyngeal squamous cell carcinoma (2000–2019)

| **Variable** | **Median survival (years, 95% CI)** | **1-year survival (95% CI)** | **5-year survival (95% CI)** | **N** | **Log-rank p** |
| --- | --- | --- | --- | --- | --- |
| Overall | 3.95 (1.89–10.11) | 0.69 (0.63–0.74) | 0.49 (0.43–0.55) | 319 | — |
| **Age group** |  |  |  |  | 0.537 |
| ≤45 years | 3.14 (1.12–.) | 0.66 (0.51–0.77) | 0.46 (0.32–0.59) | 57 |  |
| 46–74 years | 6.38 (2.31–13.68) | 0.73 (0.66–0.79) | 0.52 (0.44–0.59) | 199 |  |
| ≥75 years | 1.83 (0.71–.) | 0.60 (0.46–0.71) | 0.43 (0.30–0.56) | 63 |  |
| **Sex** |  |  |  |  | 0.148 |
| Female | 6.38 (1.81–.) | 0.70 (0.61–0.77) | 0.52 (0.43–0.60) | 146 |  |
| Male | 3.34 (1.54–9.46) | 0.69 (0.61–0.75) | 0.47 (0.38–0.54) | 173 |  |
| **Region** |  |  |  |  | 0.102 |
| Central SA | 10.10 (2.11–.) | 0.77 (0.66–0.85) | 0.55 (0.43–0.65) | 86 |  |
| Eastern SA | 4.54 (1.11–16.25) | 0.70 (0.52–0.83) | 0.49 (0.31–0.64) | 40 |  |
| Northern SA | NR (1.10–.) | 0.76 (0.52–0.89) | 0.51 (0.28–0.70) | 25 |  |
| Southern SA | 15.84 (1.81–.) | 0.78 (0.64–0.87) | 0.58(0.42–0.71) | 58 |  |
| Western SA | 1.72 (0.88–3.75) | 0.56 (0.45–0.65) | 0.39 (0.29–0.48) | 110 |  |
| **Stage at diagnosis (SEER)** |  |  |  |  | 0.148 |
| Localized | 8.44 (2.31–.) | 0.79 (0.68–0.86) | 0.57 (0.44–0.67) | 88 |  |
| Regional | 1.90 (1.14–9.46) | 0.62 (0.54–0.70) | 0.44 (0.35–0.52) | 148 |  |
| Distant | 3.52 (1.35–.) | 0.69 (0.53–0.80) | 0.49 (0.34–0.62) | 50 |  |
| Unknown | 5.74 (1.54–.) | 0.80 (0.58–0.91) | 0.55 (0.33–0.73) | 33 |  |
| **Diagnosis period** |  |  |  |  | 0.117 |
| 2000–2009 | 10.10 (2.11–.) | 0.71 (0.62–0.79) | 0.55 (0.45–0.64) | 142 |  |
| 2010–2019 | 3.00 (1.59–8.45) | 0.68 (0.60–0.74) | 0.45 (0.37–0.52) | 177 |  |

**Notes.** Mean survival values represent mean observed survival time in years with 95% confidence intervals. Survival probabilities were estimated using the Kaplan–Meier method. Log-rank tests were used to compare groups. OCC, oral cavity squamous cell carcinoma; OPC, oropharyngeal squamous cell carcinoma; CI, confidence interval.

**Supplementary Table 3.** Unstratified Cox proportional hazards regression for overall mortality among Saudi patients with oral cavity squamous cell carcinoma (2000–2019)

| **Variable** | **Unadjusted HR** | **95% CI** | **p-value** | **Adjusted HR** | **95% CI** | **p-value** |
| --- | --- | --- | --- | --- | --- | --- |
| **Age group** |  |  |  |  |  |  |
| ≤45 years (ref) | 1.00 | — | — | 1.00 | — | — |
| 46–74 years | 1.38 | 1.17–1.63 | <0.001 | 1.43 | 1.21–1.69 | <0.001 |
| ≥75 years | 1.62 | 1.33–1.97 | <0.001 | 1.66 | 1.36–2.03 | <0.001 |
| **Sex** |  |  |  |  |  |  |
| Female (ref) | 1.00 | — | — | 1.00 | — | — |
| Male | 1.47 | 1.32–1.65 | <0.001 | 1.49 | 1.33–1.68 | <0.001 |
| **Region** |  |  |  |  |  |  |
| Central SA (ref) | 1.00 | — | — | 1.00 | — | — |
| Eastern SA | 0.96 | 0.77–1.19 | 0.713 | 0.96 | 0.77–1.19 | 0.709 |
| Western SA | 1.16 | 0.98–1.37 | 0.079 | 1.15 | 0.97–1.36 | 0.120 |
| Southern SA | 1.03 | 0.88–1.20 | 0.743 | 0.99 | 0.85–1.16 | 0.927 |
| Northern SA | 1.37 | 1.05–1.78 | 0.021 | 1.26 | 0.96–1.66 | 0.099 |
| **Diagnosis period** |  |  |  |  |  |  |
| 2000–2009 (ref) | 1.00 | — | — | 1.00 | — | — |
| 2010–2019 | 1.31 | 1.16–1.47 | <0.001 | 1.37 | 1.22–1.55 | <0.001 |
| **Stage at diagnosis (SEER)** |  |  |  |  |  |  |
| Localized (ref) | 1.00 | — | — | 1.00 | — | — |
| Regional | 1.53 | 1.35–1.73 | <0.001 | 1.61 | 1.42–1.83 | <0.001 |
| Distant | 2.09 | 1.74–2.51 | <0.001 | 2.22 | 1.84–2.67 | <0.001 |
| Unknown | 1.12 | 0.88–1.43 | 0.339 | 1.14 | 0.88–1.46 | 0.321 |

**Notes:** Hazard ratios (HRs) and 95% confidence intervals (CIs) were estimated using Cox proportional hazards regression without stratification. The multivariable model included age group, sex, region, diagnosis period, and SEER summary stage at diagnosis. Analysis was restricted to Saudi nationals. HR > 1 indicates higher hazard of death relative to the reference category.

**Supplementary Table 4A.** Assessment of the proportional hazards assumption before stratification for oral cavity squamous cell carcinoma (OC-SCC)

| **Variable** | **ρ (rho)** | **χ²** | **df** | **p-value** |
| --- | --- | --- | --- | --- |
| **Age group** |  |  |  |  |
| ≤45 years (ref) | — | — | — | — |
| 46–74 years | 0.044 | 2.58 | 1 | 0.108 |
| ≥75 years | −0.028 | 1.07 | 1 | 0.301 |
| **Sex** |  |  |  |  |
| Female (ref) | — | — | — | — |
| Male | 0.013 | 0.23 | 1 | 0.628 |
| **Region** |  |  |  |  |
| Central SA (ref) | — | — | — | — |
| Eastern SA | 0.030 | 1.17 | 1 | 0.280 |
| Western SA | 0.035 | 1.73 | 1 | 0.189 |
| **Southern SA** | 0.095 | 12.61 | 1 | **<0.001** |
| Northern SA | 0.050 | 3.38 | 1 | 0.066 |
| **Diagnosis period** |  |  |  |  |
| 2000–2009 (ref) | — | — | — | — |
| 2010–2019 | −0.020 | 0.51 | 1 | 0.475 |
| **Stage at diagnosis (SEER)** |  |  |  |  |
| Localized (ref) | — | — | — | — |
| Regional | −0.162 | 32.67 | 1 | **<0.001** |
| Distant | −0.174 | 46.05 | 1 | **<0.001** |
| Unknown | −0.026 | 0.96 | 1 | 0.326 |
| **Global test** | — | **77.72** | 11 | **<0.001** |

**Notes:** Proportional hazards assumptions were evaluated using Schoenfeld residuals. Significant violations were observed for SEER summary stage (regional and distant categories) and for the Southern SA region, with a significant global test. These findings justified the application of a stage-stratified Cox model in the main analysis.

**Supplementary Table S4B**. Assessment of the proportional hazards assumption after stage-stratified Cox regression for oral cavity squamous cell carcinoma (OC-SCC)

| **Variable** | **ρ (rho)** | **χ²** | **df** | **p-value** |
| --- | --- | --- | --- | --- |
| **Region** |  |  |  |  |
| Central SA (ref) | — | — | — | — |
| Eastern SA | 0.030 | 1.15 | 1 | 0.284 |
| Western SA | 0.025 | 0.83 | 1 | 0.362 |
| Southern SA | 0.090 | 10.98 | 1 | **0.001** |
| Northern SA | 0.049 | 3.10 | 1 | 0.078 |
| **Age group** |  |  |  |  |
| ≤45 years (ref) | — | — | — | — |
| 46–74 years | 0.042 | 2.19 | 1 | 0.139 |
| ≥75 years | −0.020 | 0.55 | 1 | 0.459 |
| **Sex** |  |  |  |  |
| Female (ref) | — | — | — | — |
| Male | 0.010 | 0.12 | 1 | 0.732 |
| **Diagnosis period** |  |  |  |  |
| 2000–2009 (ref) | — | — | — | — |
| 2010–2019 | −0.023 | 0.65 | 1 | 0.420 |
| **Global test** | — | **23.08** | 8 | **0.003** |

**Notes:** Proportional hazards assumptions were reassessed after applying a stage-stratified Cox model. Stratification removed the major violation associated with stage. A residual violation persisted for the Southern region; however, this variable was not a primary exposure of interest, and its hazard ratio was close to null in the main analysis. Overall, the stratified model substantially reduced non-proportionality and improved overall model fit compared with the unstratified model.

**Supplementary Table S4C.** Assessment of the proportional hazards assumption for oropharyngeal squamous cell carcinoma (OPC-SCC)

| **Variable** | **ρ (rho)** | **χ²** | **df** | **p-value** |
| --- | --- | --- | --- | --- |
| **Age group** |  |  |  |  |
| ≤45 years (ref) | — | — | — | — |
| 46–74 years | 0.067 | 0.78 | 1 | 0.377 |
| ≥75 years | −0.064 | 0.77 | 1 | 0.381 |
| **Sex** |  |  |  |  |
| Female (ref) | — | — | — | — |
| Male | 0.146 | 3.85 | 1 | 0.050 |
| **Region** |  |  |  |  |
| Central SA (ref) | — | — | — | — |
| Eastern SA | 0.023 | 0.08 | 1 | 0.784 |
| Western SA | −0.155 | 3.81 | 1 | 0.051 |
| Southern SA | −0.039 | 0.27 | 1 | 0.602 |
| Northern SA | −0.153 | 3.93 | 1 | 0.048 |
| **Diagnosis period** |  |  |  |  |
| 2000–2009 (ref) | — | — | — | — |
| 2010–2019 | −0.000 | 0.00 | 1 | 0.997 |
| **Stage at diagnosis (SEER)** |  |  |  |  |
| Localized (ref) | — | — | — | — |
| Regional | −0.026 | 0.11 | 1 | 0.738 |
| Distant | −0.018 | 0.05 | 1 | 0.817 |
| Unknown | 0.004 | 0.00 | 1 | 0.965 |
| **Global test** | — | **16.53** | 11 | **0.123** |

**Notes:** Proportional hazards assumptions were evaluated using Schoenfeld residuals in the multivariable Cox model for OPC-SCC. The global test did not indicate a significant violation of the proportional hazards assumption. Borderline non-proportionality was observed for sex and selected regional categories, whereas stage at diagnosis did not violate the assumption.

### ****Supplementary Table S5.** Comparison of AFT model fit for OC-SCC**

| **Model** | **AIC** | **BIC** |
| --- | --- | --- |
| Weibull | 7521.6 | 7598.8 |
| Log-logistic | 7347.7 | 7424.9 |
| Log-normal | 7269.5 | 7346.7 |

**Notes:** Model fit was compared using Akaike’s Information Criterion (AIC) and Bayesian Information Criterion (BIC); lower values indicate better fit. The log-normal model demonstrated the best fit.

**2.2 Supplementary Figures**

**
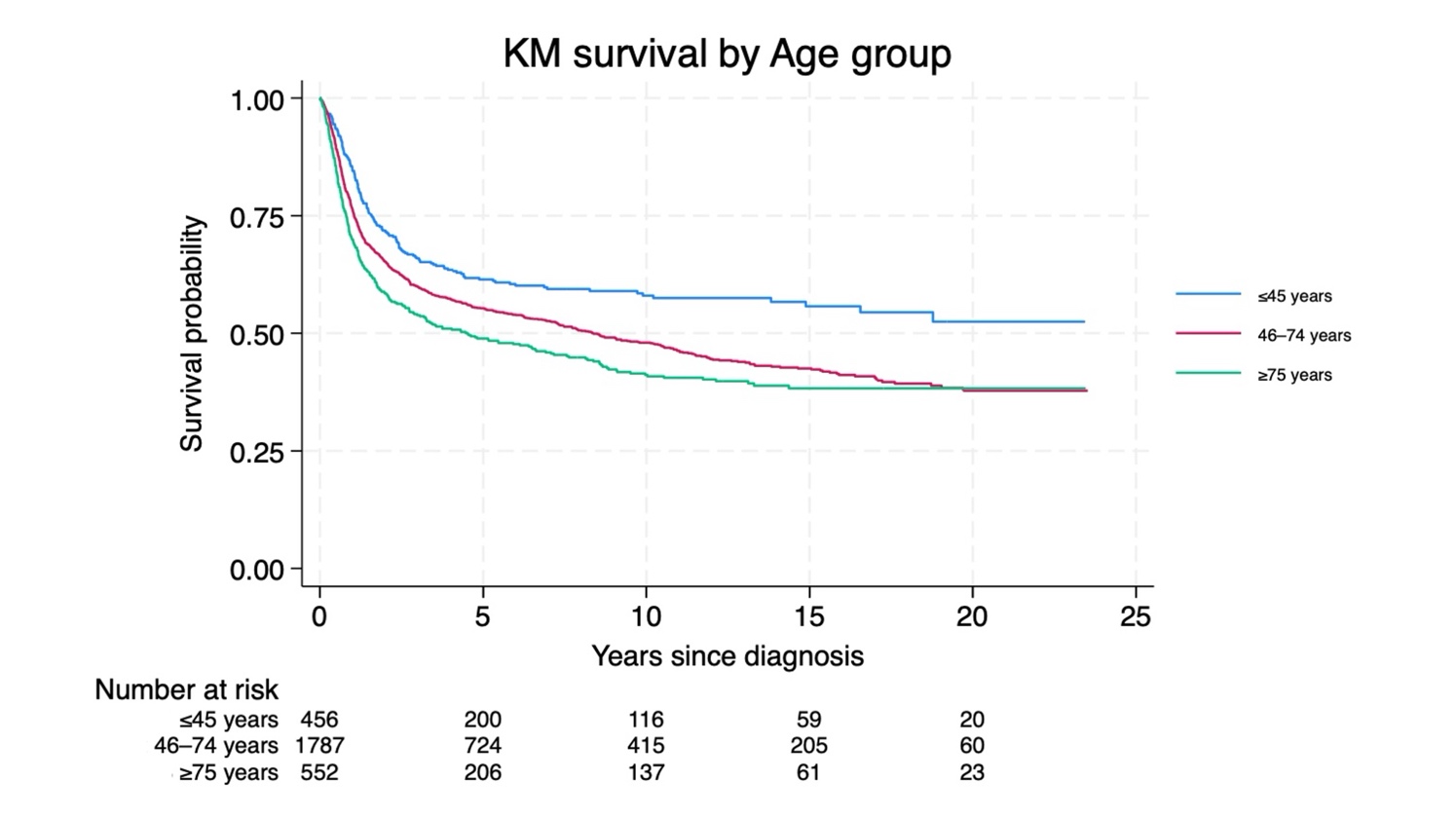
**

**Supplementary Figure 1.** Kaplan–Meier survival curves for oral cavity squamous cell carcinoma by age group

**
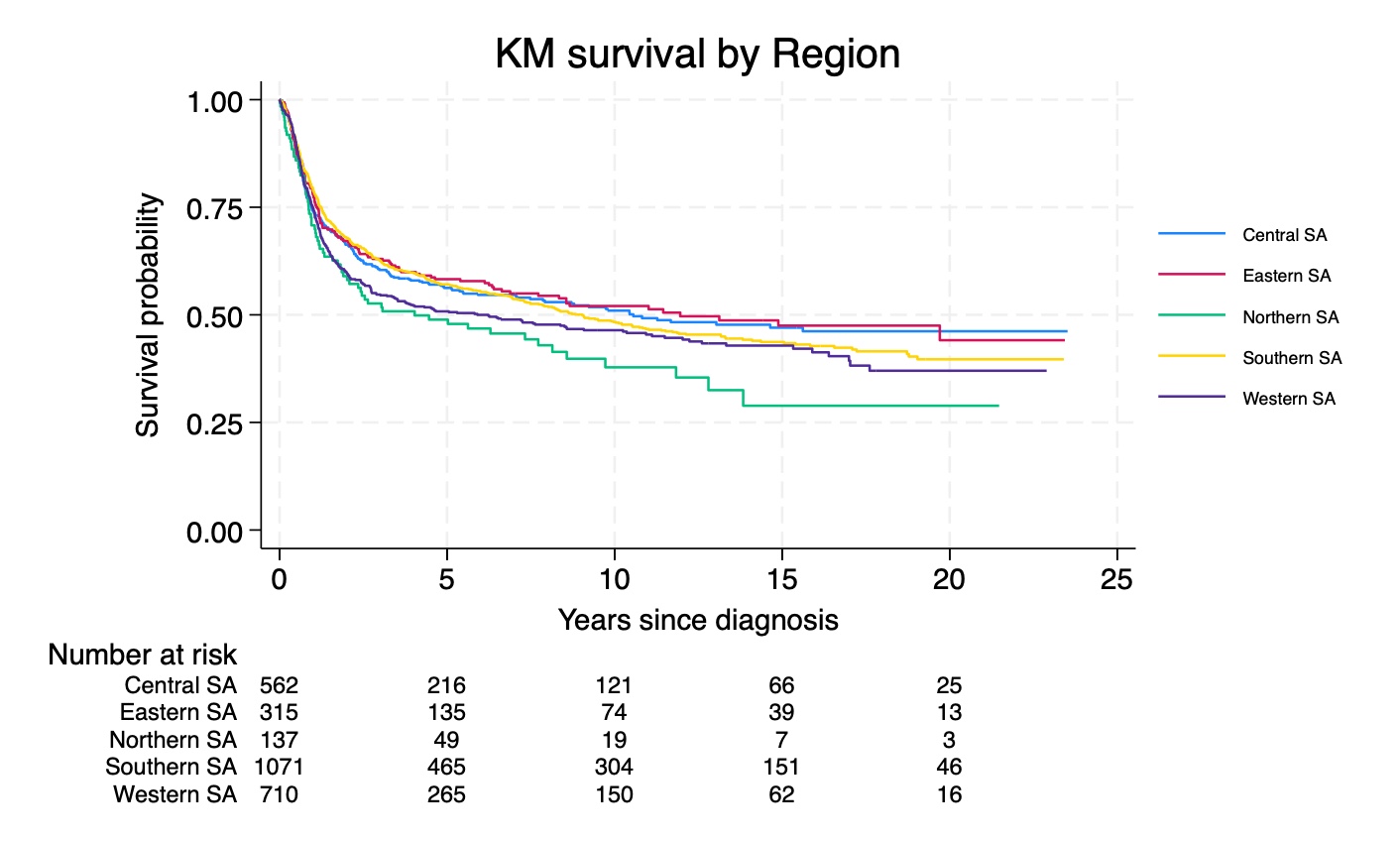
**

**Supplementary Figure 2.** Kaplan–Meier survival curves for oral cavity squamous cell carcinoma by region of residence


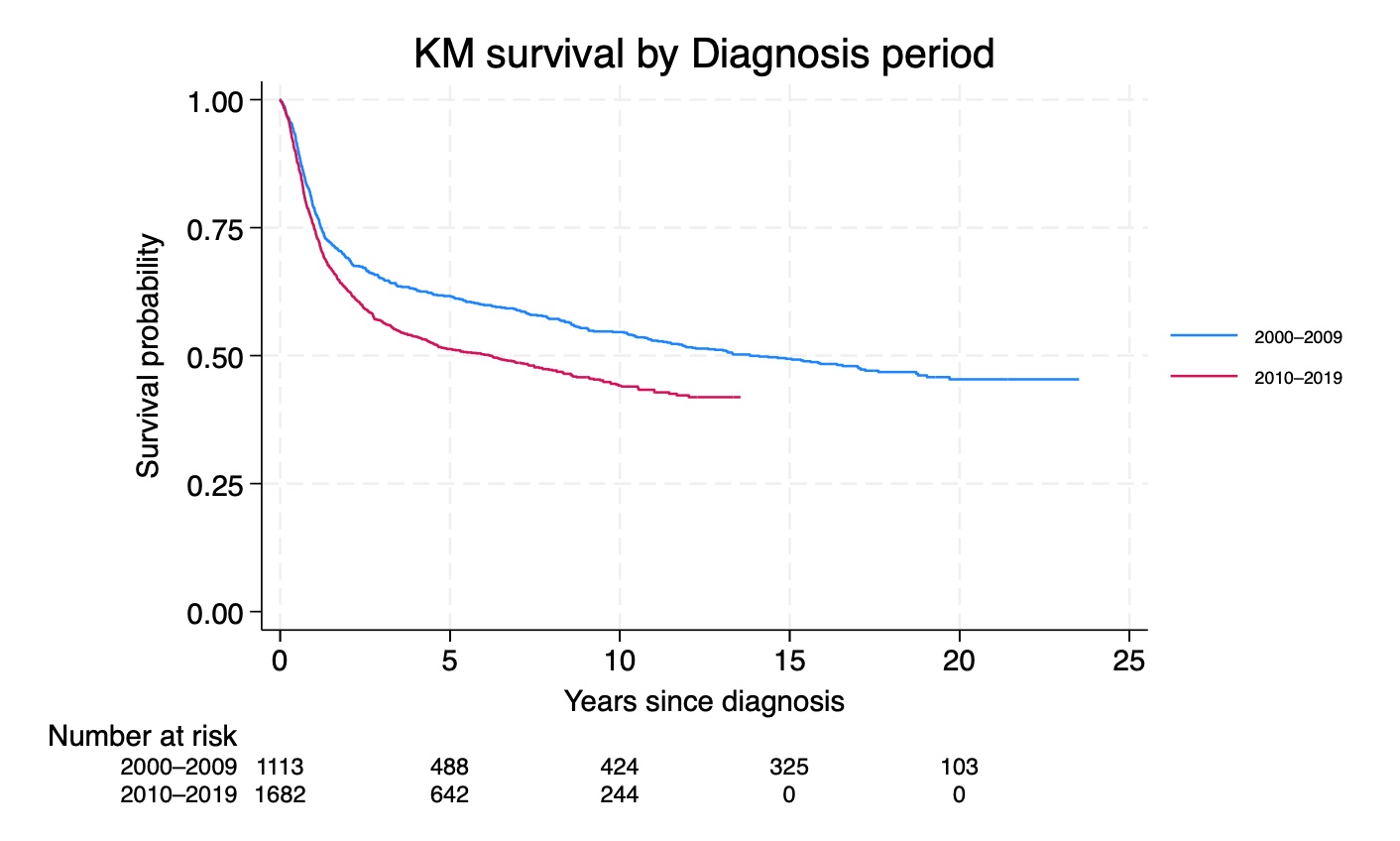


**Supplementary Figure 3.** Kaplan–Meier survival curves for oral cavity squamous cell carcinoma by diagnosis period


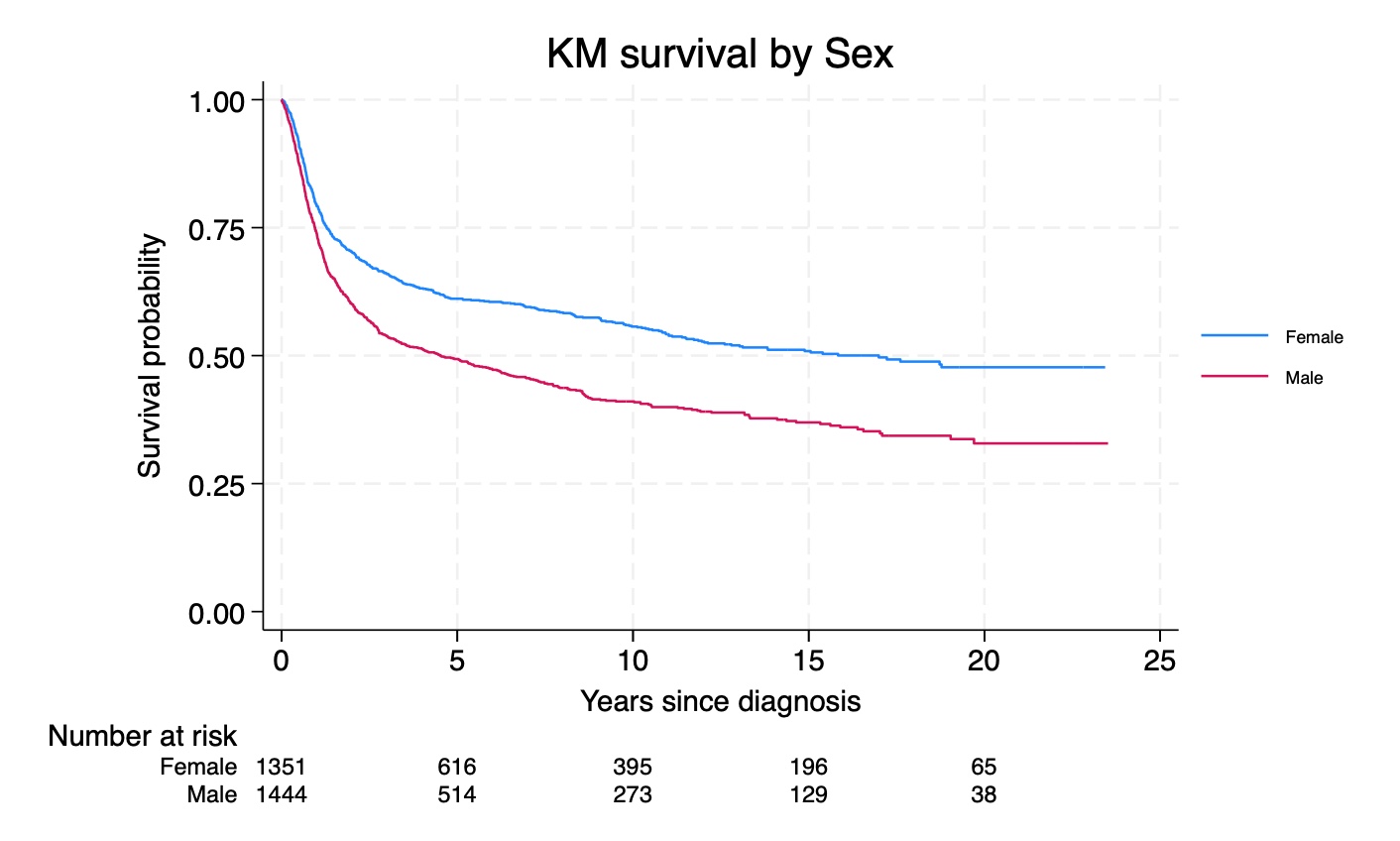


**Supplementary Figure 4.** Kaplan–Meier survival curves for oral cavity squamous cell carcinoma by sex


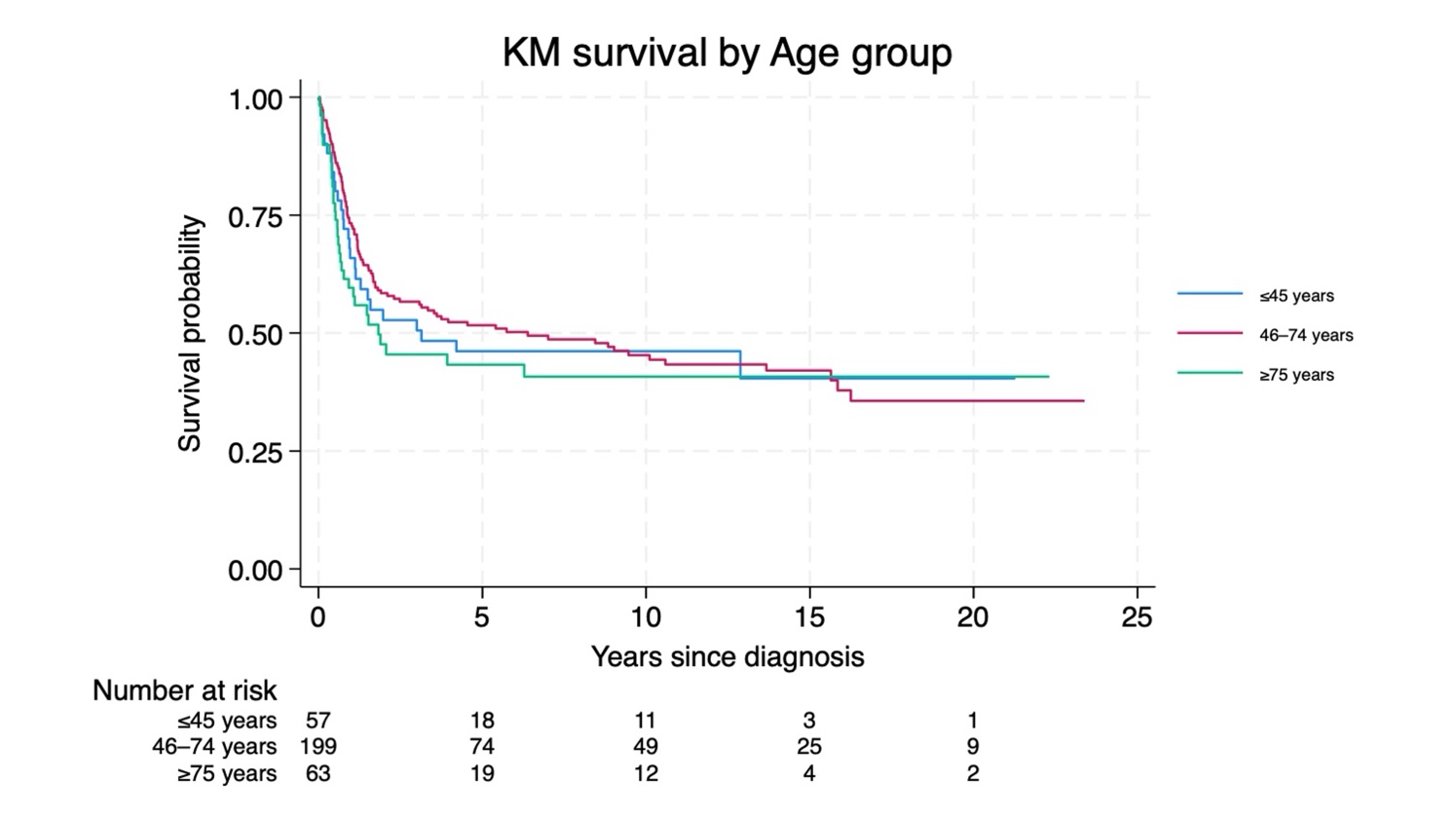


**Supplementary Figure 5.** Kaplan–Meier survival curves for oropharyngeal squamous cell carcinoma stratified by age group


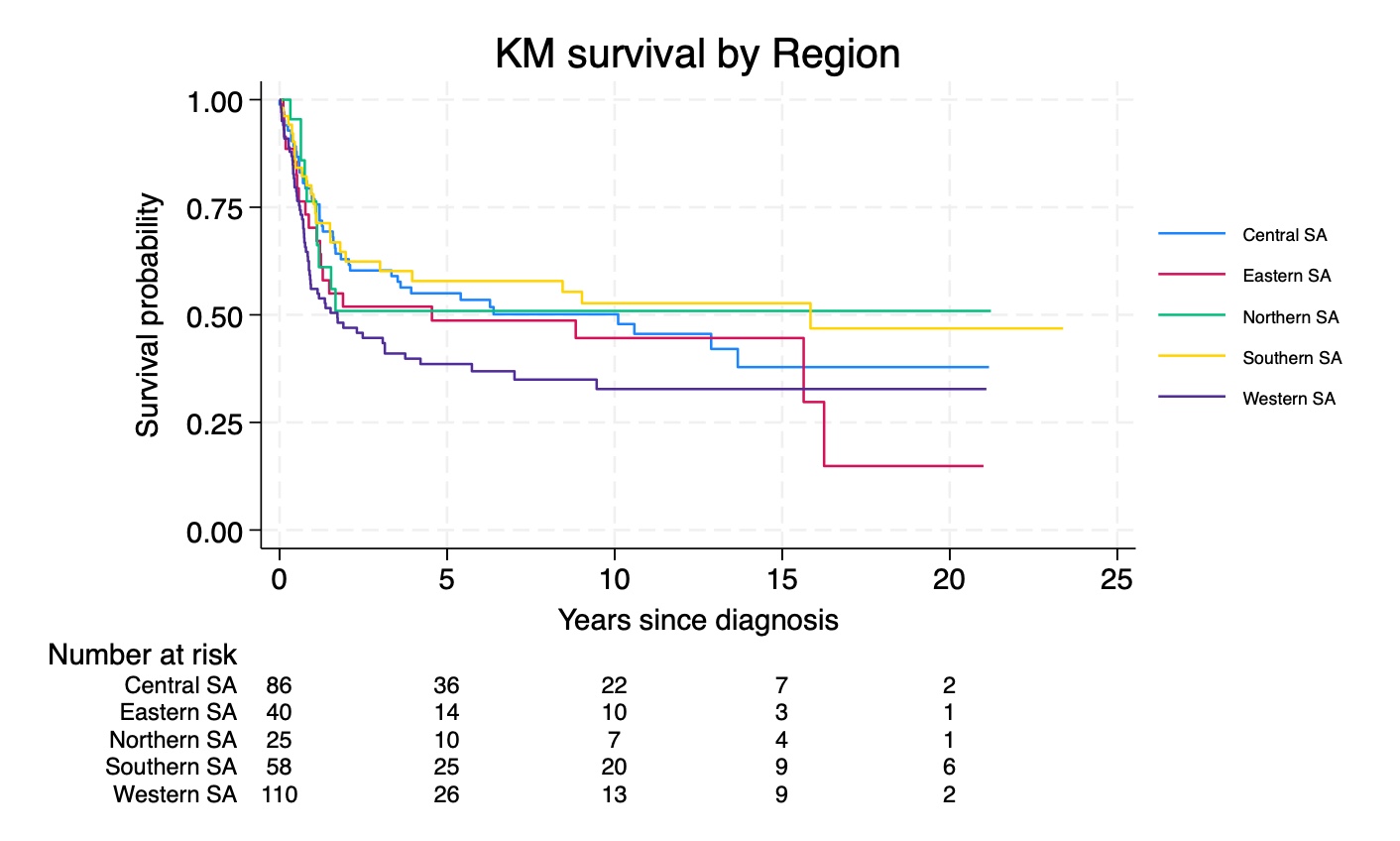


**Supplementary Figure 6.** Kaplan–Meier survival curves for oropharyngeal squamous cell carcinoma stratified by region of residence


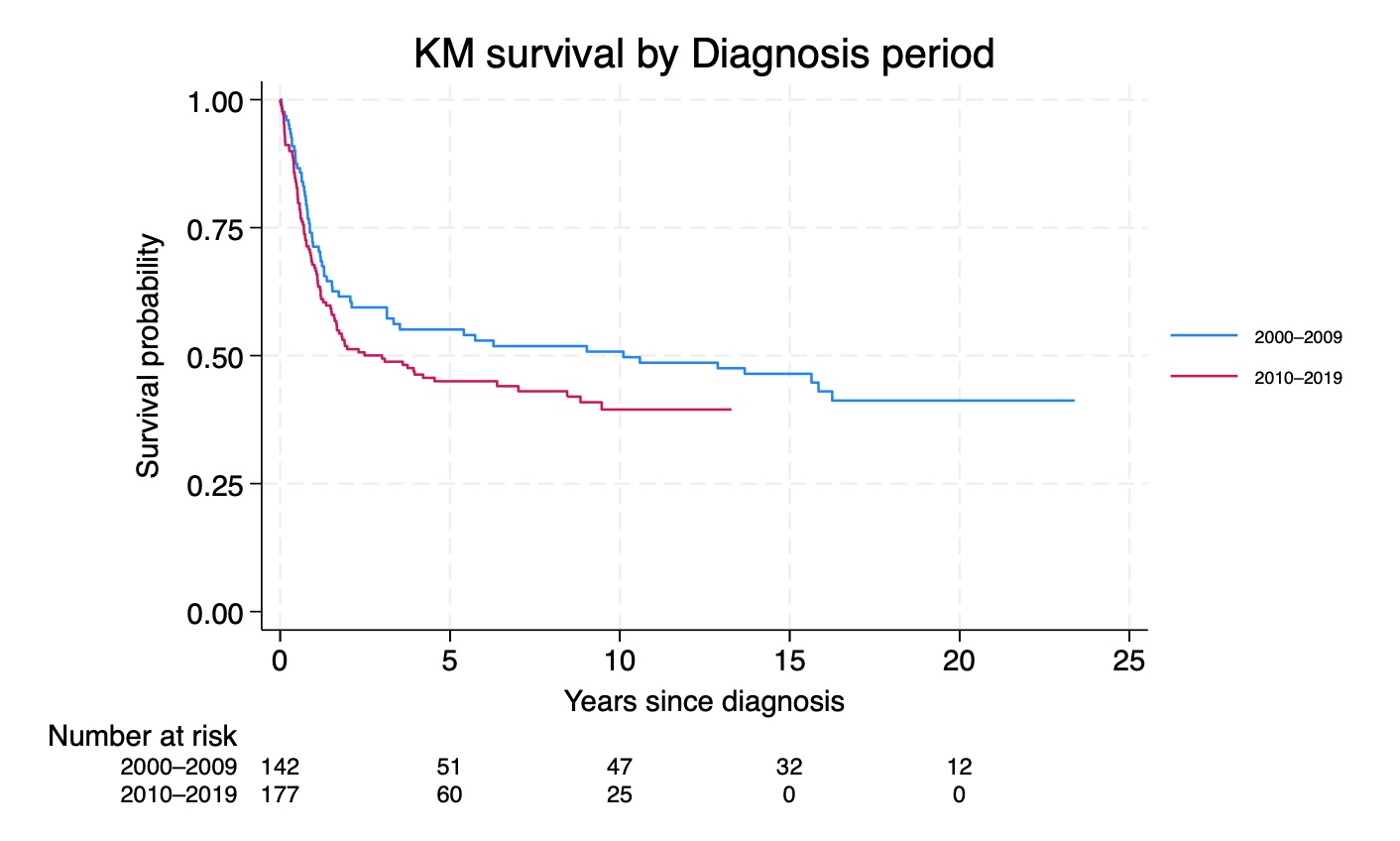
**Supplementary Figure 7.** Kaplan–Meier survival curves for oropharyngeal squamous cell carcinoma stratified by diagnosis period


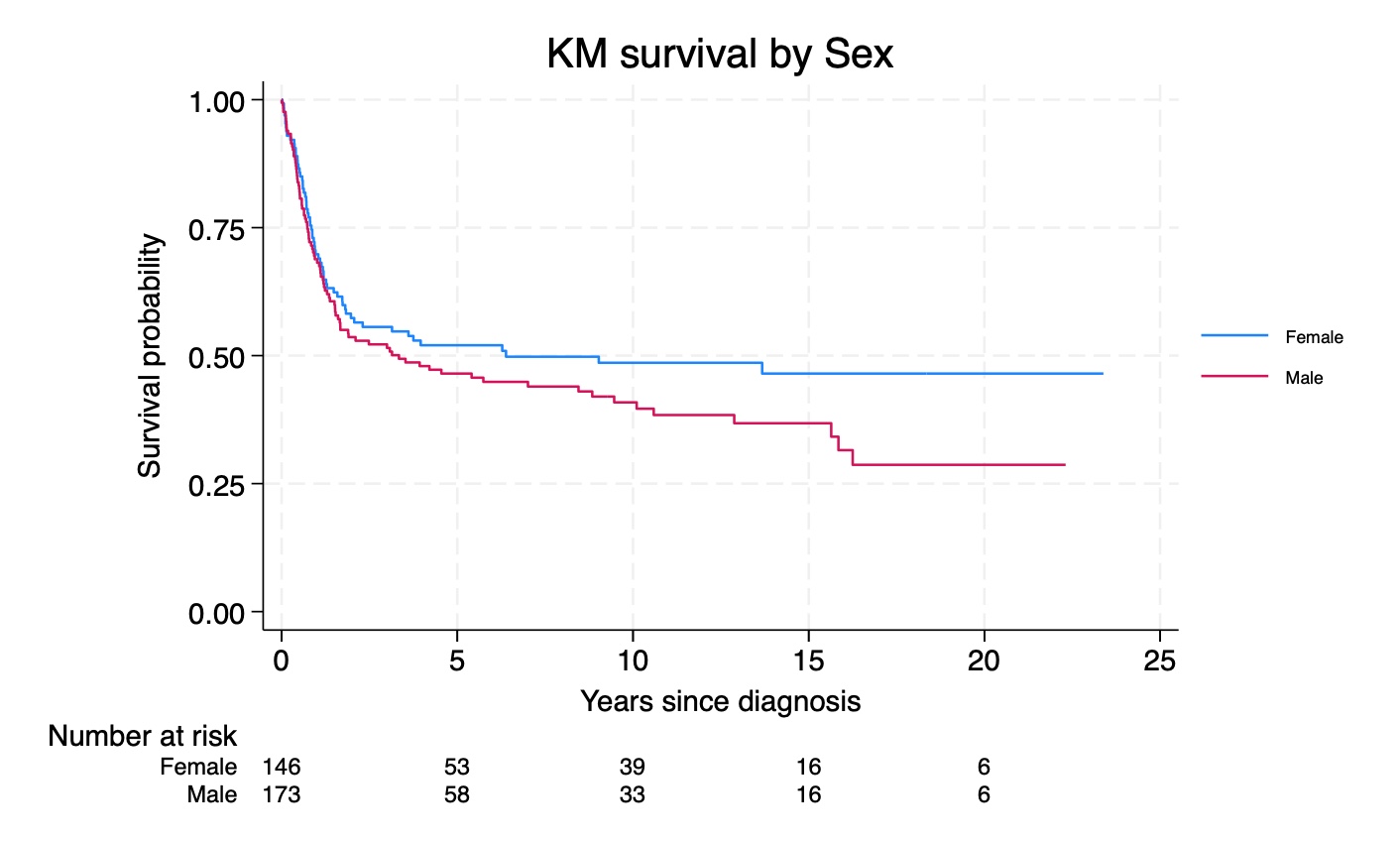


**Supplementary Figure 8.** Kaplan–Meier survival curves for oropharyngeal squamous cell carcinoma stratified by sex
